# Crystal structure and vibrational spectra of *bis*(2-isobutyrylamidophenyl)amine: a redox noninnocent ligand

**DOI:** 10.3906/kim-2106-56

**Published:** 2021-09-12

**Authors:** Emrah ASLANTATAR, Savita K. SHARMA, Omar VILLANUEVA, Cora E. MACBETH, İlkay GÜMÜŞ, Hakan ARSLAN

**Affiliations:** 1 Department of Chemistry, Faculty of Arts and Science, Mersin University, Mersin, Turkey; 2 Department of Chemistry, Emory University, 1515 Dickey Drive, Atlanta, USA; 3 School of Science and Technology, Georgia Gwinnett College, Lawrenceville, USA; 4 Advanced Technology Research and Application Center, Mersin University, Mersin, Turkey

**Keywords:** Redox noninnocent ligand, single crystal structure, Hirshfeld surface analysis, infrared spectrum, ab initio calculations, Hartree-Fock method, density functional theory method

## Abstract

The molecular structure of *bis*(2-isobutyrylamidophenyl)amine (H_3_L^NNN^) has been determined from single-crystal X-ray diffraction data. The crystal packing of H_3_L^NNN^ is governed by the N–H···O and C–H···O hydrogen-bonding and C–H···π stacking interactions between the vicinal molecules. The intermolecular interactions in the crystal structure of H_3_L^NNN^ have been also examined via Hirshfeld surface analysis and fingerprint plots. The Hirshfeld surface analysis showed that the important role of N–H···O and C–H···π interactions in the solid-state structure of H_3_L^NNN^. The molecular structure, vibrational frequencies, and infrared intensities of H_3_L^NNN^ were computed by ab initio HF and DFT (B3LYP, B3PW91, and BLYP) methods using the 6–31G(d,p) basis set. The computed theoretical geometric parameters were compared with the corresponding single crystal structure of H_3_L^NNN^. The harmonic vibrations calculated for the title compound by the B3LYP method are in good agreement with the experimental IR spectral data. The theoretical vibrational spectrum of the H_3_L^NNN^ compound was interpreted through potential energy distributions using the SQM Version 2.0 program. The performance of the used methods and the scaling factor values were calculated with PAVF Version 1.0 program.

## 1. Introduction

In recent years, a large amount of work has been devoted to the study of transition metal redox processes for electron transfer processes due to the importance of the electron transfer process in the development of industrial useful catalysts [1–6]. The coordination of ligands to metal ions is one way of attenuating metal-based redox processes. During a transition metal-mediated redox process, an electron can be accepted by or released from a metal center. When redox noninnocent ligands are coordinated to transition metal ions, the ligand can also participate in electron transfer processes. So, ligand design is very important. Recently, we have focused on the design and catalytic activity of Co(II) complexes formed with tridentate redox-innocent compounds as a ligand [7,8]. In the light of these findings and the continuation of our research studies on the tripodal ligand system, our team produced and characterized a number of substituted tridentate ligands and their metal complexes [8–11]. We demonstrate that our synthesized transition metal complexes by using *bis*(2–isobutyrylamidophenyl)amine as the tripodal redox noninnocent ligand are capable of catalytic oxidation reactions using dioxygen. This ligand system has two *N*-amidate donor atoms and one amido donor and supports coordinatively unsaturated metal centers with open coordination sites available for small molecule binding. This ligand stabilizes both mononuclear and dinuclear cobalt(II) complexes able to catalytically oxidize PPh_3_ to Ph_3_PO with much better catalytic efficiencies than those previously observed for cobalt(II) complexes in the presence of excess dioxygen under ambient conditions. Performing these reactions with the large substrate to catalyst loading ratio (500:1) gives maximum turnover numbers of 185 and 345 mol product/mol catalyst for the cobalt(II) complexes. In addition, the most recent application of this ligand system derivatived with different functional groups is the ability for catalytic C-H amination to form indolines from aryl azides by cobalt(II) complexes of them [12]. In that, the study of redox behavior of ligands is important for the development of new catalysts. The most suitable markers for determining the redox behavior of the ligand are the C-X (X = C, N, O, S, Se) stretching vibration modes and bond distances. If the ligand is redox noninnocent, the coordination of the metal decreases C-X bond distances, and C-X stretching vibrations shifts to lower frequencies via radical parts formed on the ligand skeletal. The experimental vibrational spectra are accurately reproduced by the calculations, which show that C-C, C-N, and C-X vibration modes are extensively mixed with other modes, and thus unsuitable to work as vibrational markers [13]. Therefore, in this study, we aim that learn more information about the structure of the redox noninnocent ligands due to their role in catalytic processes. To achieve this aim, we selected *bis*(2-isobutyrylamidophenyl)amine as a sample redox noninnocent ligand. We have calculated the structural parameters and vibration modes of H_3_L^NNN^ in the ground state to distinguish the fundamentals from the structural parameters and experimental vibrational frequencies by using the HF [14], B3LYP [15,16], BLYP [15,16], and B3PW91 [15,17], with the standard 6-31G(d,p) basis set. The calculated structural parameters and vibration modes were analyzed and compared with obtained experimental results. In the current work, we also investigated the relative performance of B3LYP, BLYP, and B3PW91 methods, as well as of HF for comparison, at the 6-31G(d,p) level taking as a test compound *bis*(2-isobutyrylamidophenyl)amine. On the other hand, the role of intermolecular interactions of *bis*(2-isobutyrylamidophenyl)amine has been analyzed through single-crystal structure studies, and these intermolecular interactions in the single crystal structure of *bis*(2-isobutyrylamidophenyl) amine have been visualized via Hirshfeld surface analysis and fingerprint plots.

## 2. Experimental

### 2.1. Instrumentation

^1^H and ^13^C NMR were obtained on a Bruker Avance III 400 MHz Ultrashield Plus Biospin spectrometer. The deuterated solvent DMSO-*d*_6_ was used as purchased. FT-IR spectra were recorded on a Perkin Elmer Spectrum 100 series FT-IR spectrometer in KBr disc and were reported in cm^−1^ units (4000–400 cm^−1^; number of scans: 250; resolution: 1 cm^−1^). X-ray diffraction studies were carried out in the X-ray Crystallography Laboratory at Emory University on a Bruker Smart 1000 CCD diffractometer. Mass spectra were recorded on an Agilent 6460 series LC-MS/MS trap with electrospray ionization (ESI) source and triple quadrupole ion trap mass analyzer by direct infusion and ESI operated in the positive and negative mode in Advanced Technology Research and Application Center, Mersin University, Mersin, Turkey. Acetonitrile: water (0.1% formic acid) (95:5, %) was used as mobile phase and 2 μL of the sample injected at 0.3 mL/min flow rate [Column: Zorbax Eclipse XDB-C18 (4.6 mm I.D. × 50 mm L., 1.8 μm)].

### 2.2. Synthesis

**Figure 1:**
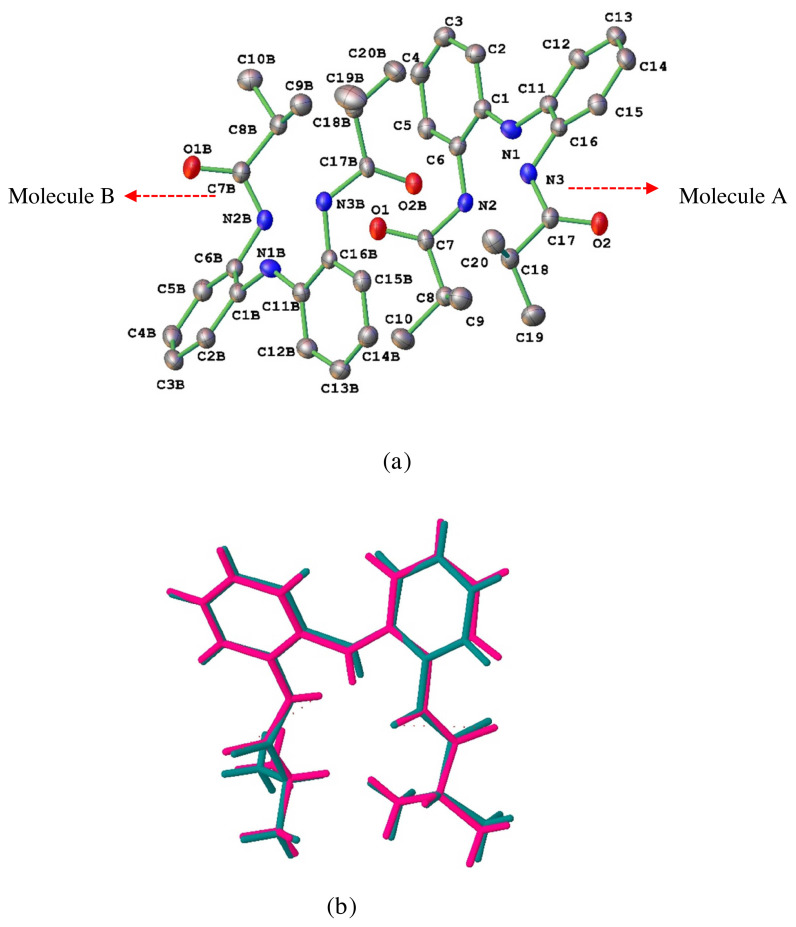
(a) Crystal structure of *bis*(2-isobutyrylamidophenyl)amine. Thermal ellipsoids are shown at the 50% probability level and hydrogen atoms have been removed for clarity. (b) Overlay diagram of two independent molecules.

**Figure 2:**
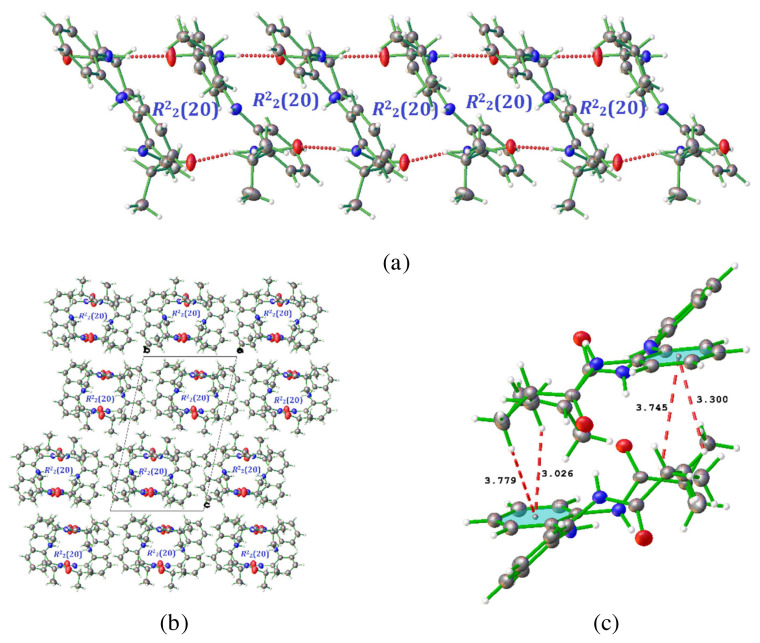
The formation of *R*^2^_2_(20) synthon generated through N–H···O hydrogen bonds along the crystallographic (a) [010] direction, (b) [100] direction, (c) C-H···π stacking interactions.

**Figure 3:**
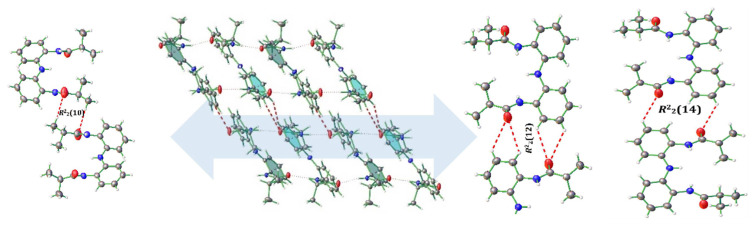
Consecutive the formation of *R*^2^_2_(10), *R*^2^_2_(12), and *R*^2^_2_(14) synthon generated through C–H···O hydrogen bonds.

**Figure 4:**
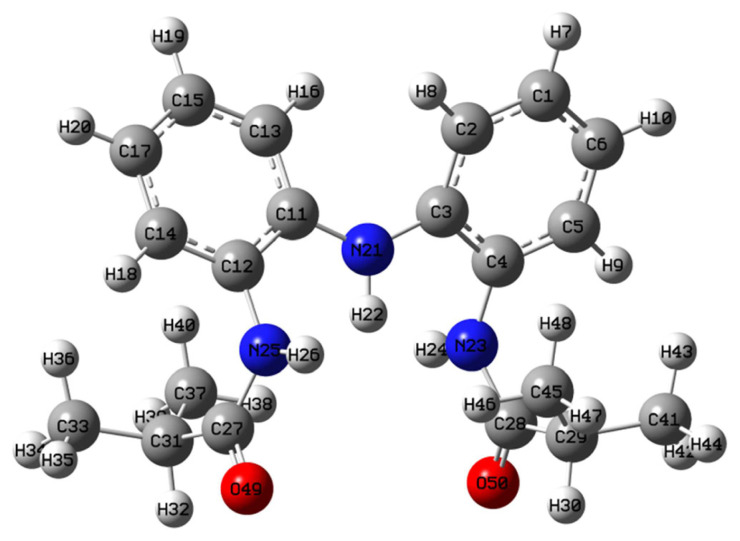
The optimized geometry of H_3_L^NNN^ calculated at B3LYP/6-31G(d,p) level.

**Figure 5:**
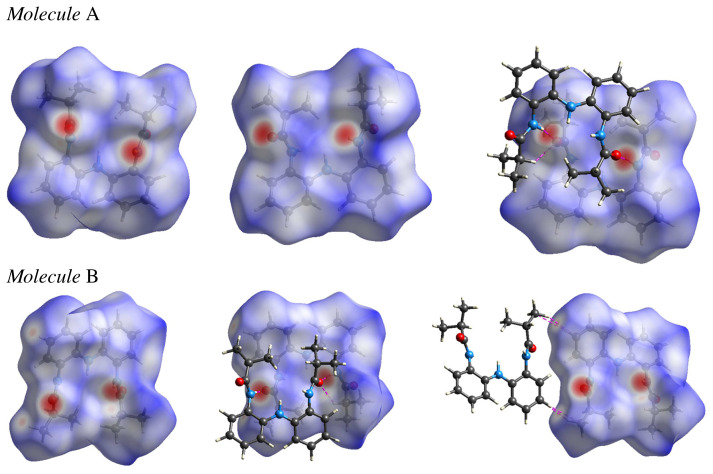
*d*_norm_ Hirshfeld surface and *d*_norm_ Hirshfeld surface surrounded by one neighboring molecule associated with close contacts of molecules A and B.

**Figure 6:**
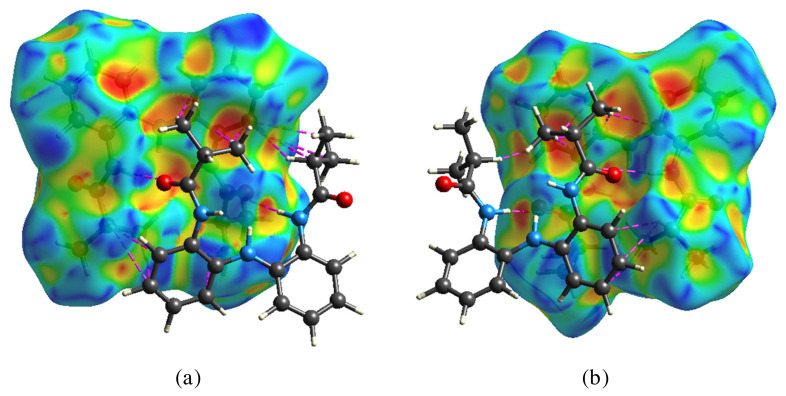
Hirshfeld surface of molecules A (left) and B (right) mapped with shape index function.

2–Nitroaniline, 1–fluoro–2–nitrobenzene, and Pd/C were obtained from Sigma Aldrich and used as received. All other chemicals were purchased from different suppliers and used without further purification. *Bis*(2–nitrophenyl)amine and *bis*(2–aminophenyl)amine are prepared by using the given literature procedures [18,19]. Preparation of compound H_3_L^NNN^ was carried out as in Scheme, adapting the reported procedure (Figures 1S–6S) [8]. Yield: 92 %. ^1^H NMR (400 MHz, DMSO-*d*_6_, δ, ppm): 9.38 (s, 2H, NH(CO)), 7.39 (dd, 2H, Ar-H), 7.05 (td, 2H, Ar-H), 6.92 (m, 4H, Ar-H), 6.86 (s, 1H, NH), 2.62 (m, 2H, CH), 1.07 (s, 12H, CH_3_).^13^C NMR (100 MHz, DMSO-*d*_6_, δ, ppm): 175.54, 137.27, 128.78, 125.49, 125.35, 120.81, 119.01, 34.30, 19.31. LC-MS (+ESI, *m/z*): 340.2 [M+H]^+^, 322.1, 270.1, 252.2, 200.3, 183.2, 106.9.

### 2.3. Theoretical studies

Theoretical calculations were made with the Gaussian 03W program [20]. The molecular structure of H_3_L^NNN^ in the ground state was optimized by using BLYP, B3LYP, B3PW91, and HF methods with 6-31G(d,p) basis set. The vibrational frequencies were also computed with the same methods and basis set. The frequency values computed at these levels contain known systematic errors [21]. These differences can be corrected using scaling factor values of 0.8992, 0.9614, 1.0072, and 0.9573 for HF, B3LYP, BLYP, and B3PW91, respectively [22–27]. The scaled quantum mechanical procedure has been widely used in the identification of the vibrational bands of IR and RAMAN spectrums [28]. The vibrational modes were assigned using SQM Version 2.0 program on the principle of potential energy distribution analysis [29]. The performance of the methods used was quantitatively characterized using the PAVF Version 1.0 program [30,31].

### 2.4. Hirshfeld surface analysis

Analysis of Hirshfeld surfaces and their associated 2D fingerprint plots of H_3_L^NNN^ were computed by using CrystalExplorer 3.1 [32]. The Hirshfeld surfaces are mapped with different properties such as shape index, *d*_norm_, etc. The *d*_norm_ is normalized contact distance, defined in terms of *d*_e_, *d*_i_, and the vdW radii of the atoms. The combination of *d*_e_ and *d*_i_ in the form of a 2D fingerprint plot displays a summary of intermolecular contacts in the crystal.

## 3. Results and discussion

The synthesis of the title compounds involves the reaction of an isobutyryl chloride with *bis*(2–aminophenyl)amine in dichloromethane in the presence of triethylamine. The compound was recrystallized by layering hexane onto a concentrated CH_2_Cl_2_ solution of the product and characterized by ^1^H NMR, ^13^C NMR, LC-MS/MS, FT–IR, and X–ray single-crystal diffraction method. All data obtained are consistent with the expected structure.

### 3.1. Molecular geometry

The molecular structure of *bis*(2–isobutyrylamidophenyl)amine was confirmed by the single crystal X-ray structure studies (Figure 1a). For H_3_L^NNN^, data collection and refinement are summarized in Table 1. Bond lengths, angles, and hydrogen bond details of the title compound are also presented in Tables 2–4, respectively (Tables 1S and 2S).

The bond distance of the carbonyl groups in the title compound is typical for the double-bond character, C7–O1 = 1.228(3) Å, C17–O2 = 1.231(3) Å. However, the CN bond distances for the investigated compound are all shorter than the average single CN bond distance of 1.48 Å, being N1–C1 = 1.394(3) Å, N1–C11 = 1.391(3) Å, N3–C16 = 1.423(3) Å, N3–C17 = 1.356(3) Å, N2–C6 = 1.428(3) Å, and N2–C7 = 1.351(3) Å. These evidences indicate a partial electron delocalization within the C(O)–NH–Ph–NH–Ph–NH–C(O) fragment. These obtained results are in agreement with the expected delocalization in H_3_L^NNN^ and confirmed by C7–N2–C6 = 125.9(2)°, C1–N1–C11 = 130.0(2)° and C17–N3–C16 = 124.5(2)° showing a *sp*^2^ hybridization on the N1, N2 and N3 atoms. All other bond distances are within the expected ranges [33].

In the crystal structure of the title compound, the molecules are connected by intermolecular hydrogen bonds: N2- H2A···O1B*^i^*, with H···O 1.89 Å, N-H···O 176°, N3-H3A···O2B*^ii^*, with H···O 1.99 Å, N-H···O 171°, N2B-H2BA···O1*^ii^*, with H···O 1.93 Å, N-H···O 176°, and N3B-H3BA···O2*^iii^*, with H···O 2.00 Å, N-H···O 167° [Symmetry codes: (*i*) 1+*x*, +*y*, +*z*; (*ii*) *x*, *y*, *z*; (*iii*) −1+*x*, +*y*, +*z*] (Figures 2a–2c and 3).

The unit cell of H_3_L^NNN^ contains two independent molecules in the asymmetric unit, represented as A and B in Figure 1a and these molecules are virtually identical conformation as you can see in Figure 1b. Molecules A and B interact via strong N-H···O (Table 4) hydrogen bonds between amide hydrogen atom as strong hydrogen bond donor and carbonyl oxygen atom as strong hydrogen bond acceptor in the asymmetric unit. Moreover, the N-H···O hydrogen bonds continue infinitely and lead to the formation of infinite dimeric *R*^2^_2_(20) synthons (Figure 2a). These dimeric synthons in the asymmetric unit expand along the crystallographic [010] direction. The formation of dimeric synthons in H_3_L^NNN^ is also supported by additional bifurcated C-H···π interactions between phenyl rings and aliphatic hydrogen atoms (Figures 2b and 2c).

The infinite chain occurring via N–H···O H-bonds and C-H···π stacking interactions is layered by consecutive three different types of C–H···O dimeric motifs [*R*^2^_2_(10), *R*^2^_2_(12) and *R*^2^_2_(14)], providing an overall 3D-multilayered structure. The *R*^2^_2_(10) dimeric motif is due to the interaction between aliphatic hydrogen atoms and carbonyl group oxygen atom of two neighboring molecules. On the other hand, the *R*^2^_2_(12) and *R*^2^_2_(14) dimeric motifs occur between aryl ring hydrogen atoms and carbonyl group oxygen atom of two neighboring molecules (Figure 3).

The point group symmetry of the molecular structure of the H_3_L^NNN^ compound is *C*_S_. We have performed a full structural optimization of the H_3_L^NNN^ compound and the optimized geometrical parameters calculated by HF and DFT methods (Table 5, Figure 4). In addition, we have compared the experimental geometric parameters with the calculated one and we found that the calculated bond distances and angles show good agreement with experiment one. The best agreement with the experimental values was obtained for the HF and B3LYP methods for bond lengths and bond angles, respectively. The largest difference between calculated and experimental bond distances and angles are 0.042 Å and 5.95°, respectively, for DFT/B3LYP-6-31G(d,p) method. From the calculated values, it has been found that most of the optimized bond distances are slightly larger than the experimental bond distances since the calculations are for isolated molecules in the gas phase and the experimental results are for the solid-state molecules [34–39]. Although there are minor differences between experimental and theoretical values, the calculated geometric parameters represent a good approximation and are the basis for calculating other parameters such as vibrational frequencies and thermodynamic properties.

The computed thermodynamic parameters (such as thermal energy, specific heat capacity, dipole moment, rotational constants, entropy, and zero-point vibrational energy) of H_3_L^NNN^ by all used methods are listed in Table 6. The structure optimization and zero-point vibrational energy of H_3_L^NNN^ in HF, BLYP, B3LYP, and B3PW91/6-31G(d,p) are 282.8046, 256.6969, 264.7935, and 265.3738 kcal/mol, respectively. The global minimum energy obtained for structure optimization of H_3_L^NNN^ is −1092 a.u. for the B3LYP method. The minimum energy becomes −1085 a.u. for HF. The difference in the amount of energy between the methods is ca. 7 a.u. only.

### 3.2. Vibrational assignments

FT-IR spectrum of the title compound is given in Figure 6S. Table 7 lists the vibration frequencies obtained using B3LYP calculations along with an approximate description of each of the experimental frequencies and normal modes. The other calculations (HF, B3PW91, and BLYP) were given as supplementary materials (Tables 3S and 4S).

The title compound has 50 atoms; thus, it gives 144 (3n − 6) normal modes of vibration. All vibration modes are active in both infrared and Raman spectrums. Generally, the theoretical vibrational frequencies are higher than the experimental ones, because of anharmonicity of the incomplete treatment of electron correlation and of the use of finite one-particle basis set [37,40,41]. Therefore, these wavenumbers must be scaled by a proper scale factor and, in this research study, we have used the scaling factor values for HF, B3LYP, BLYP, and B3PW91 as 0.8992, 0.9614, 1.0072, and 0.9573, respectively. The identification of the vibration bands was made using the SQM 2.0 program [29] and the animation option of the GaussView 5.0 program [27]. All experimental vibrational frequencies are in good agreement with the theoretical ones. According to Table 7, experimental vibrational frequencies are in better agreement with the scaled vibrational frequencies and are found to have a good correlation for B3LYP than BLYP, B3PW91, and HF methods.

In the heterocyclic compounds, *ν*_N-H_ vibration occurs in the region 3500–3000 cm^−1^. The IR band appearing at 3406, 3398, and 3367 cm^−1^ is assigned to the *ν*_N-H_ stretching mode of vibrations. These vibration modes are computed at 3451, 3404, and 3404 cm^−1^ for the B3LYP method. The differences between experimental and computed *ν*_N-H_ stretching modes are about 45, 6, and 37 cm^−1^ (DFT-B3LYP/6-31G(d,p). These striking discrepancies can come from the formation of intermolecular hydrogen bonding with N-H. This interpretation is verified with *ν*_C=O_ stretching vibration mode. The differences between experimental (1695 and 1679 cm^−1^) and computed (1707 and 1704 cm^−1^) *ν*_C=O_ are about 12 and 25 cm^−1^, respectively. It can be easily observed in the correlation graphics of the computed and experimental frequencies of H_3_L^NNN^. Also, all the obtained results are agree with the single crystal structure of H_3_L^NNN^. It is clear that, in the crystal structure, the molecules are connected by intermolecular H-bonds: N3-H3A···O2B, N2–H2A···O1B, N2B–H2BA···O1, and N3B–H3BA···O2 (Figures 2a–2c).

The characteristic CH stretching vibration modes ν_CH_ of the aromatic structure of the H_3_L^NNN^ compound are expected to appear in the frequency range 3100–3000 cm^−1^ [42–45]. Although eight vibrational modes are calculated in the 3100–3000 cm^−1^ range, the ν_CH_ stretching vibration modes of H_3_L^NNN^ were assigned to four bands observed in the IR spectrum. This difference between the calculated and observed vibration band numbers is due to the overlapping of the aromatic ν_CH_ stretching vibrational frequencies. The first two bands (3118 and 3115 cm^−1^) are symmetric ν_CH_ stretching vibration modes and the others (3099 and 3059 cm^−1^) are asymmetric ν_CH_ stretching vibration modes of the aromatic structure [46].

For the assignments of methyl group frequencies, 39 fundamental vibration modes can be associated with methyl groups. Twelve stretchings, nine deformations, six rockings, five umbrellas, and seven torsion vibration modes have designated the motion of the methyl group. The methyl symmetric and asymmetric stretching frequencies are observed at 3035, 3001, 2966, 2964, 2938, and 2929 cm^−1^ in the IR spectrum of the title compound. The minor differences between observed and calculated asymmetric stretching vibrational modes may be due to strong C-H···π interaction which are observed in the crystal form (Figures 2a–2c, Table 7). The observed bands at 1382 and 1357 cm^−1^ are attributed to methyl umbrella vibration modes [24]. The bands observed at 1109, 1049, and 902 cm^−1^ are assigned to the rocking vibration modes of the methyl group.

The bands due to the δ_CH_ in-plane aromatic ring bending vibration mode interacting with the ν_CC_ stretching vibration mode are observed in the region 1608-950 cm^−1^ [47]. ϒ_CH_ vibration modes are strongly coupled vibrations and occur in the region 964–721 cm^−1^. All the δ_CH_ and ϒ_CH_ bending vibration modes of the CH group have been identified and they are given in Table 7.

The identification of CN stretching vibration modes ν_CN_ is difficult because of the mixing of the other vibration modes. However, we solved this problem by the GaussView Version 3.0 and the SQM Version 2.0 programs [29]. Therefore, the CN stretching vibration modes are clearly identified and assigned in this research. Some of the vibration bands appearing between 1421 and 1049 cm^−1^ are assigned as CN stretching vibration modes (Table 7). All the obtained results agree with the literature [48].

Generally, the C=C stretching vibration modes are seen in the region of 1430–1650 cm^−1^ for aromatic compounds [49–52]. The C-C stretching vibration modes of the title compound are observed at 1608, 1597, 1579, 1568, 1527, 1490, 1436, and 1421 cm^−1^. All bands lie in the expected range when compared to the literature values [46]. The C-C-C in-plane bending vibration modes are observed between 879 and 520 cm^−1^ and the ϒ_CC_ vibration modes are calculated between 737 and 466 cm^−1^.

A general better performance of B3LYP versus the other methods can be quantitatively characterized by using the root mean square values, the mean absolute percentage error, and the coefficients of correlation (*r*) between the observed and computed vibration frequencies. All these obtained data were computed in this study by the PAVF Version 1.0 program [30] according to Scott and Radom. The coefficients of correlation values for all DFT methods were greater than 0.9993 and they are very close to those reported in the literature [43–55].

The root mean square errors of the experimental and calculated vibration bands are found to be 13.49, 14.57, 15.48, and 31.06 for B3LYP, B3PW91, BLYP, and HF methods, respectively. These obtained results indicate that the fundamental frequencies computed by B3LYP, B3PW91, and BLYP methods for the H_3_L^NNN^ compound show good agreement with the experimental values. Especially, B3LYP has the best agreement. A small difference between the calculated and experimental vibrational modes is also observed. These small differences due to the formation of inter- and intramolecular hydrogen bonding. In addition, we note that the theoretical calculations belong to the gaseous phase and the experimental results belong to the solid phase [37].

We also computed the optimal scaling factors, which are crucial for vibrational spectral identification, using the PAVF 1.0 program [30]. Only single-uniform scaling factors were calculated without accounting for different vibrations. The single-uniform scaling factor values obtained are 0.9606, 0.9895, 0.9576, and 0.9034 for the B3LYP, BLYP, B3PW91, and HF methods, respectively. These obtained scaling factor values are very close to those recommended by Scott and Radom [22] for the same levels of theory (0.9614, 1.0072, 0.9573, and 0.8992, respectively). Thus, for future vibrational spectral predictions for unknown derivatives of H_3_L^NNN^, one can recommend scaling factors 0.9606, 0.9895, 0.9576, and 0.9034 for the B3LYP, BLYP, B3PW91, and HF methods, respectively.

### 3.3. Hirshfeld surface analysis

Hirshfeld surface analysis for molecules A and B in the asymmetric unit of H_3_L^NNN^ was calculated by using the program CrystalExplorer 3.1 [32]. The Hirshfeld surface was helped to distinguish the similarities and differences between the symmetry-independent molecules A and B present in the asymmetric unit. The Hirshfeld surfaces of H_3_L^NNN^ were investigated to clarify the nature of the intermolecular interactions and are illustrated in Figures 5, 6a and 6b showing the surfaces that have been mapped over a *d*_norm_ and shape index functions. The surfaces are shown as transparent to allow visualization of the molecular moiety, in a similar orientation for the molecules, around which they were calculated. In the *d*_norm_ Hirshfeld surface, contacts with distances equal to the sum of the van der Waals radii are represented as white regions and the contacts with distances shorter than and longer than van der Waals radii are shown as red circles and blue areas, respectively [56,57].

In front and back *d*_norm_ surfaces of molecule A, a total of four dark red spots were observed; these dark red spots are for the short N–H···O hydrogen bonds between molecules A and B. Moreover, there is one smaller red spot corresponding to weaker C-H···O interactions. On the other hand, in front and back *d*_norm_ surfaces of molecule B, a total of seven red spots were observed; the four dark red spots in these surfaces are for the short N–H···O H-bonds between molecules A and B, and the other three (light red spots) in front *d*_norm_ surfaces are for C···H interactions (also recognizable on Hirshfeld surface mapped with shape index function, Figures 6a and 6b) between phenyl carbon atom of molecule B and phenyl/methyl hydrogen atom vicinal molecule and C-O···H interactions between carbonyl O atom of molecule A and aliphatic H atom of molecule B. This indicates that these interactions play a very important role in the formation of crystals.

The analysis about C···H interactions of the molecules A and B was done using the Hirshfeld surface shape index (Figures 6a and 6b). C···H/H···C interactions mainly responsible for the molecular packing in the supramolecular structure and represent C-H···π interactions. On the Hirshfeld surface mapped with shape index function, one can notice both hollow orange (π···H) and bulging blue regions (H···π) corresponding to C–H···π interactions [58,59].

The 2D fingerprint plots obtained from the Hirshfeld surface analysis for each independent molecule in the asymmetric unit provide quantitative information for the individual intermolecular atom-atom contacts of a molecule in the crystal environment. The fingerprint plots can be decomposed to highlight particular atoms pair close interactions in the compound. The decomposed fingerprint plots for the two crystallographically independent molecules A and B are shown in Figure 7S. For both molecules A and B, the H···H interactions have the highest contribution of the total Hirshfeld surface with 60.9 and 61.7%, respectively, and the contribution from the H···H contact is 0.8% more for molecule B compared to molecule A. Despite the high share of H···H interactions, the role of these interactions in the stabilization of crystal structure is quite small in importance because this interaction is between the same species. In both fingerprints plots for molecules A and B, the two sharp spikes responsible for the strong N–H···O H-bond formation were observed. These contributions are almost similar with a difference of 0.4% for both molecules. On the other hand, the wings regions were observed which correspond to the C···H interactions, attributed to C–H···π interactions, in both fingerprints plots of molecules A and B. The contribution from the C···H contact is 1.2% more for molecule A in comparison with molecule B.

## 4. Conclusion

The molecular structure of *bis*(2-isobutyrylamidophenyl) amine has been solved by the single-crystal X-ray diffraction studies. The crystal packing of H_3_L^NNN^ shows N–H···O, C–H···O, and C–H···π inter-molecular interactions. The N–H···O interactions between molecules are among the strongest reported interactions for H_3_L^NNN^. The Hirshfeld surfaces analysis has been used for more investigation of intermolecular interactions as a driving force for the crystal structure of the H_3_L^NNN^ compound formation has been demonstrated. In addition, the relative contribution of intermolecular interactions in H_3_L^NNN^ is analyzed by fingerprint plots of the Hirshfeld surface. The ground state geometries were optimized using the B3LYP, BLYP, B3PW91, and HF methods. The vibration modes were also computed with these methods. The theoretical vibrational modes are in good agreement with its observed FT-IR spectrum of H_3_L^NNN^. Optimal uniform scaling factors were also computed for the H_3_L^NNN^ compound. The three hybrid functions can be equally successful for vibrational spectrum predictions for the H_3_L^NNN^ compound type derivatives. Taking small variations of the scaling factors into account for the derivatives of H_3_L^NNN^, one can recommend scaling factors of 0.9606, 0.9895, 0.9576, and 0.9034 for the B3LYP, BLYP, B3PW91, and HF methods, respectively, for future vibrational spectral assignments for unknown compounds of this class.

## Supporting information

**Crystal structure and vibrational spectra of** ***bis*** **(2-isobutyrylamidophenyl)amine: a redox noninnocent ligand**

Emrah ASLANTATAR ^1^, Savita K. SHARMA ^2^, Omar VILLANUEVA ^3^, Cora E. MACBETH ^2^, Ilkay GUMUS ^1,4^ and Hakan ARSLAN ^1,2,4,*^

^1^Department of Chemistry, Faculty of Arts and Science, Mersin University, Mersin, Turkey

^2^Department of Chemistry, Emory University, 1515 Dickey Drive, Atlanta, USA

^3^ School of Science and Technology, Georgia Gwinnett College, Lawrenceville, USA

^4^Advanced Technology Research and Application Center, Mersin University, Mersin, Turkey

^*^ Corresponding author, e-mail: hakan.arslan@mersin.edu.tr[Table t8-turkjchem-45-6-1933]

**Table t8-turkjchem-45-6-1933:** 

Index	Page
**Figure**	
Figure 1S. ^1^H NMR spectra of H_3_L^NNN^ in DMSO-*d*_6_.	2
Figure 2S. ^13^C NMR spectra of H_3_L^NNN^ in DMSO-*d*_6_.	3
Figure 3S. COSY-NMR spectra of H_3_L^NNN^ in DMSO-*d*_6_.	4
Figure 4S. HMQC-NMR spectra of H_3_L^NNN^ in DMSO-*d*_6_.	5
Figure 5S. LC-MS spectra of H_3_L^NNN^.	6
Figure 6S. FT-IR spectrum of H_3_L^NNN^.	7
Figure 7S. 2D fingerprint plots of molecules A and B.	8

**Table**	

Table 1S. All bond lengths for H_3_L^NNN^.	9
Table 2S. All bond angles for H_3_L^NNN^.	10
Table 3S. Vibrational wavenumbers obtained for H_3_L^NNN^.	11
Table 4S. Optimized and experimental geometries of H_3_L^NNN^ in the ground state.^*^	14

Figure 1S ^1^H NMR spectra of H_3_L^NNN^ in DMSO-*d*_6_.

Figure 2S ^13^C NMR spectra of H_3_L^NNN^ in DMSO-*d*_6_.

Figure 3S COSY-NMR spectra of H_3_L^NNN^ in DMSO-*d*_6_.

Figure 4S HMQC-NMR spectra of H_3_L^NNN^ in DMSO-*d*_6_.

Figure 5S LC-MS spectra of H_3_L^NNN^.

Figure 6S FT-IR spectrum of H_3_L^NNN^.

Figure 7S 2D fingerprint plots of molecules A and B.

**Table 1S:** All bond lengths for H_3_L^NNN^.*

Atom	Atom	Length (Å)	Atom	Atom	Length (Å)
C1	C2	1.394(3)	C1B	C2B	1.391(3)
C1	C6	1.400(3)	C1B	C6B	1.403(3)
C1	N1	1.394(3)	C1B	N1B	1.395(3)
C2	C3	1.383(3)	C2B	C3B	1.380(3)
C3	C4	1.372(3)	C3B	C4B	1.383(4)
C4	C5	1.387(3)	C4B	C5B	1.387(3)
C5	C6	1.384(3)	C5B	C6B	1.377(3)
C6	N2	1.428(3)	C6B	N2B	1.429(3)
C7	C8	1.509(3)	C7B	C8B	1.512(3)
C7	N2	1.351(3)	C7B	N2B	1.335(3)
C7	O1	1.228(3)	C7B	O1B	1.227(3)
C8	C9	1.523(3)	C8B	C9B	1.531(3)
C8	C10	1.512(3)	C8B	C10B	1.520(3)
C11	C12	1.399(3)	C11B	C12B	1.387(3)
C11	C16	1.408(3)	C11B	C16B	1.397(3)
C11	N1	1.391(3)	C11B	N1B	1.395(3)
C12	C13	1.375(3)	C12B	C13B	1.380(3)
C13	C14	1.380(4)	C13B	C14B	1.379(3)
C14	C15	1.386(3)	C14B	C15B	1.384(3)
C15	C16	1.377(3)	C15B	C16B	1.377(3)
C16	N3	1.423(3)	C16B	N3B	1.434(3)
C17	C18	1.504(3)	C17B	C18B	1.502(3)
C17	N3	1.356(3)	C17B	N3B	1.362(3)
C17	O2	1.231(3)	C17B	O2B	1.232(3)
C18	C19	1.519(3)	C18B	C19B	1.519(4)
C18	C20	1.538(3)	C18B	C20B	1.516(3)

* The atom-numbering scheme of the molecular structure is given in Figure 1a.

**Table 2S:** All bond angles for H_3_L^NNN^.*

Atom	Atom	Atom	Angle (°)	Atom	Atom	Atom	Angle (°)
C2	C1	C6	118.2(2)	C2B	C1B	C6B	118.1(2)
C2	C1	N1	123.8(2)	C2B	C1B	N1B	123.6(2)
N1	C1	C6	117.9(2)	N1B	C1B	C6B	118.2(2)
C3	C2	C1	120.6(2)	C3B	C2B	C1B	120.7(2)
C4	C3	C2	120.9(3)	C2B	C3B	C4B	120.9(3)
C3	C4	C5	119.3(2)	C3B	C4B	C5B	118.9(3)
C6	C5	C4	120.4(3)	C6B	C5B	C4B	120.6(3)
C1	C6	N2	118.4(2)	C1B	C6B	N2B	119.1(2)
C5	C6	C1	120.6(3)	C5B	C6B	C1B	120.8(2)
C5	C6	N2	121.0(2)	C5B	C6B	N2B	120.1(2)
N2	C7	C8	115.2(2)	N2B	C7B	C8B	116.4(2)
O1	C7	C8	122.9(2)	O1B	C7B	C8B	122.3(3)
O1	C7	N2	121.9(3)	O1B	C7B	N2B	121.3(3)
C7	C8	C9	110.3(2)	C7B	C8B	C9B	109.6(2)
C7	C8	C10	112.0(2)	C7B	C8B	C10B	111.1(2)
C10	C8	C9	110.4(2)	C10B	C8B	C9B	111.9(2)
C12	C11	C16	118.2(2)	C12B	C11B	C16B	117.8(2)
N1	C11	C12	123.4(2)	C12B	C11B	N1B	123.9(2)
N1	C11	C16	118.4(2)	N1B	C11B	C16B	118.2(2)
C13	C12	C11	120.6(2)	C13B	C12B	C11B	121.2(3)
C12	C13	C14	121.2(2)	C14B	C13B	C12B	120.7(3)
C13	C14	C15	118.7(2)	C13B	C14B	C15B	118.6(3)
C16	C15	C14	121.2(2)	C16B	C15B	C14B	121.1(3)
C11	C16	N3	118.8(2)	C11B	C16B	N3B	119.4(2)
C15	C16	C11	120.1(2)	C15B	C16B	C11B	120.6(3)
C15	C16	N3	121.1(2)	C15B	C16B	N3B	119.9(2)
N3	C17	C18	115.7(2)	N3B	C17B	C18B	115.7(2)
O2	C17	C18	122.4(2)	O2B	C17B	C18B	122.5(2)
O2	C17	N3	121.8(2)	O2B	C17B	N3B	121.7(3)
C17	C18	C19	111.2(2)	C17B	C18B	C19B	108.2(2)
C17	C18	C20	108.3(2)	C17B	C18B	C20B	112.2(2)
C19	C18	C20	111.9(2)	C20B	C18B	C19B	111.2(2)
C11	N1	C1	130.0(2)	C11B	N1B	C1B	128.9(2)
C7	N2	C6	125.9(2)	C7B	N2B	C6B	122.7(2)
C17	N3	C16	124.5(2)	C17B	N3B	C16B	123.7(2)

* The atom-numbering scheme of the molecular structure is given in Figure 1a.

**Table 3S:** Vibrational wavenumbers obtained for H_3_L^NNN^.

NO	SYM	Exp.	Calculated															

			B3LYP	B3LYP × SF	B3LYP × SF	IR_INT	B3PW91	B3PW91 × SF	B3PW91 × SF	IR_INT	BLYP	BLYP × SF	BLYP × SF	IR_INT	HF	HF × SF	HF × SF	IR_INT
1	A	3406	3589	3451	3448	63.43	3595	3441	3442	65.74	3455	3480	3418	59.54	3918	3523	3540	59.25
2	A	3398	3541	3404	3401	0.47	3565	3412	3413	0.21	3414	3439	3378	1.00	3819	3434	3451	3.15
3	A	3367	3541	3404	3401	35.13	3565	3412	3413	43.65	3414	3439	3378	17.78	3819	3434	3451	70.83
4	A	3118	3232	3107	3105	5.33	3236	3098	3099	6.28	3150	3173	3117	7.90	3394	3052	3066	6.42
5	A	3118	3223	3099	3096	1.42	3231	3093	3094	2.13	3141	3163	3108	1.54	3388	3046	3061	3.05
6	A	3115	3215	3091	3088	2.10	3226	3089	3089	1.26	3134	3156	3101	0.80	3379	3038	3053	1.88
7	A	3115	3214	3090	3088	33.69	3225	3087	3088	27.28	3133	3156	3100	41.86	3378	3038	3052	47.05
8	A	3099	3201	3077	3075	21.96	3212	3075	3076	18.48	3120	3143	3087	27.26	3363	3024	3038	28.11
9	A	3099	3201	3077	3075	2.03	3212	3075	3076	1.69	3120	3142	3087	4.77	3363	3024	3038	4.27
10	A	3059	3189	3066	3063	5.58	3201	3064	3065	3.96	3106	3128	3074	7.40	3348	3011	3025	6.11
11	A	3059	3188	3065	3063	3.25	3200	3063	3064	3.58	3106	3128	3073	3.27	3348	3010	3025	3.10
12	A	3035	3141	3019	3017	63.58	3156	3021	3022	41.68	3064	3086	3031	51.72	3290	2958	2972	113.33
13	A	3035	3140	3019	3017	1.78	3156	3021	3022	4.12	3063	3085	3031	3.88	3289	2958	2972	3.93
14	A	3001	3135	3014	3012	17.46	3151	3016	3017	11.14	3054	3076	3022	28.39	3278	2947	2961	21.70
15	A	3001	3135	3014	3012	8.52	3151	3016	3017	16.22	3054	3076	3022	11.58	3278	2947	2961	4.95
16	A	2966	3120	2999	2997	41.55	3138	3004	3005	18.86	3041	3063	3009	19.35	3255	2927	2941	9.09
17	A	2966	3120	2999	2997	6.51	3138	3004	3005	29.74	3041	3063	3009	40.07	3255	2927	2941	99.92
18	A	2964	3112	2992	2990	4.55	3131	2998	2999	5.52	3032	3054	3000	5.95	3252	2924	2938	0.10
19	A	2964	3112	2992	2989	51.28	3131	2998	2998	46.73	3032	3054	3000	56.67	3251	2924	2937	20.71
20	A	2958	3072	2954	2951	7.76	3080	2948	2949	6.91	2983	3005	2952	9.89	3246	2919	2933	0.78
21	A	2958	3072	2954	2951	0.32	3080	2948	2949	0.96	2983	3005	2952	1.28	3246	2919	2932	4.96
22	A	2938	3051	2934	2931	0.02	3060	2929	2930	29.56	2977	2998	2945	38.30	3193	2871	2885	0.02
23	A	2938	3051	2934	2931	31.13	3060	2929	2930	0.33	2977	2998	2945	0.27	3193	2871	2885	28.19
24	A	2929	3049	2931	2928	52.46	3058	2927	2928	1.18	2973	2995	2942	53.82	3190	2868	2882	2.30
25	A	2929	3049	2931	2928	1.60	3058	2927	2928	53.51	2973	2995	2942	3.53	3190	2868	2882	66.66
26	A	1695	1776	1707	1706	495.87	1792	1715	1716	506.21	1691	1703	1673	417.05	1947	1750	1759	651.67
27	A	1679	1773	1704	1703	139.36	1789	1713	1713	162.12	1689	1701	1671	131.10	1944	1748	1757	260.70
28	A	1608	1662	1597	1596	2.25	1675	1604	1604	1.63	1594	1606	1578	1.54	1811	1629	1636	1.80
29	A	1597	1649	1585	1584	200.24	1662	1591	1591	226.88	1583	1594	1566	222.46	1799	1617	1625	10.42
30	A	1579	1637	1574	1573	111.12	1646	1576	1577	102.99	1572	1583	1555	63.67	1787	1607	1615	277.73
31	A	1568	1628	1565	1564	0.69	1640	1570	1571	1.84	1562	1574	1546	0.22	1774	1596	1603	0.50
32	A	1527	1574	1513	1512	486.94	1582	1515	1515	523.32	1516	1527	1500	421.46	1708	1535	1543	362.88
33	A	1490	1534	1474	1473	84.39	1539	1473	1474	109.30	1491	1502	1476	18.29	1670	1502	1509	149.17
34	A	1472	1530	1471	1469	5.69	1522	1457	1457	17.52	1490	1501	1475	28.97	1646	1480	1487	41.41
35	A	1472	1529	1470	1469	30.21	1521	1456	1457	34.92	1485	1496	1470	1.33	1644	1479	1486	23.16
36	A	1458	1522	1463	1462	1.55	1515	1450	1451	3.89	1485	1496	1469	3.47	1636	1471	1478	14.49
37	A	1458	1521	1463	1461	29.05	1515	1450	1451	22.61	1478	1488	1462	41.75	1636	1471	1478	1.08
38	A	1454	1513	1455	1454	0.02	1509	1444	1445	0.14	1476	1487	1461	3.66	1628	1464	1471	0.68
39	A	1454	1513	1455	1453	0.74	1504	1440	1440	0.56	1475	1486	1460	22.67	1628	1464	1471	0.02
40	A	1447	1506	1448	1447	0.08	1504	1440	1440	0.00	1469	1480	1454	0.85	1624	1461	1467	0.15
41	A	1446	1506	1448	1447	2.97	1501	1436	1437	89.63	1469	1480	1454	1.68	1624	1460	1467	8.60
42	A	1446	1505	1447	1445	0.44	1497	1433	1433	1.43	1457	1467	1442	0.61	1621	1457	1464	0.03
43	A	1436	1498	1440	1439	101.95	1497	1433	1433	11.75	1451	1462	1436	85.73	1620	1457	1464	56.00
44	A	1421	1474	1417	1416	31.67	1476	1413	1414	38.65	1423	1434	1409	31.29	1615	1453	1459	119.87
45	A	1406	1440	1385	1383	41.14	1444	1382	1383	63.68	1398	1408	1383	17.78	1600	1438	1445	22.98
46	A	1392	1440	1384	1383	38.26	1442	1380	1380	41.67	1397	1407	1383	22.99	1582	1423	1429	18.13
47	A	1382	1435	1380	1378	2.34	1429	1368	1368	8.87	1384	1394	1369	15.11	1562	1404	1411	13.09
48	A	1382	1433	1378	1377	4.49	1427	1366	1367	17.02	1384	1394	1369	5.68	1561	1404	1411	11.79
49	A	1357	1411	1356	1355	2.07	1403	1343	1344	3.79	1373	1383	1358	1.14	1541	1386	1392	0.59
50	A	1357	1411	1356	1355	6.47	1403	1343	1344	12.75	1373	1382	1358	4.97	1540	1385	1392	3.15
51	A	1325	1375	1322	1321	107.77	1384	1325	1325	65.21	1333	1343	1319	124.57	1486	1337	1343	246.25
52	A	1307	1360	1308	1307	0.29	1371	1312	1312	3.25	1321	1330	1307	0.77	1485	1335	1342	19.40
53	A	1307	1360	1308	1307	6.14	1361	1303	1303	315.67	1319	1328	1305	17.27	1482	1333	1339	0.32
54	A	1305	1357	1305	1304	113.42	1356	1298	1299	91.52	1317	1327	1304	4.51	1482	1333	1339	0.65
55	A	1305	1354	1302	1301	65.15	1353	1295	1295	29.47	1310	1320	1297	43.29	1476	1328	1334	0.75
56	A	1288	1348	1296	1295	5.51	1352	1294	1294	109.60	1309	1319	1296	35.86	1475	1326	1333	291.37
57	A	1286	1338	1286	1285	303.84	1339	1282	1282	43.84	1297	1306	1283	14.77	1447	1301	1308	348.05
58	A	1286	1335	1283	1282	2.94	1339	1281	1282	29.46	1288	1297	1274	11.10	1442	1296	1303	5.29
59	A	1263	1333	1281	1280	164.54	1334	1277	1277	0.06	1270	1279	1257	363.41	1407	1265	1271	22.05
60	A	1263	1321	1270	1269	0.52	1329	1272	1273	5.73	1264	1273	1250	171.17	1381	1242	1248	3.99
61	A	1249	1288	1238	1237	16.47	1296	1241	1241	16.04	1246	1255	1233	18.33	1331	1197	1203	13.58
62	A	1213	1279	1230	1229	3.52	1291	1236	1236	2.15	1231	1240	1219	1.29	1324	1191	1196	18.55
63	A	1195	1227	1179	1178	4.51	1229	1177	1177	3.43	1186	1195	1174	3.67	1315	1183	1188	0.81
64	A	1126	1222	1174	1174	3.38	1222	1170	1170	3.02	1180	1189	1168	4.59	1303	1172	1177	2.91
65	A	1109	1197	1150	1150	16.20	1195	1144	1144	7.24	1162	1170	1149	5.15	1297	1166	1171	8.61
66	A	1109	1197	1150	1149	5.34	1195	1144	1144	20.00	1160	1168	1148	16.70	1296	1166	1171	3.56
67	A	1109	1191	1145	1144	1.83	1188	1137	1138	1.08	1159	1167	1147	2.60	1226	1102	1107	44.60
68	A	1109	1189	1143	1142	3.53	1186	1136	1136	2.87	1159	1167	1147	7.41	1222	1099	1104	47.38
69	A	1097	1133	1090	1089	10.33	1144	1095	1095	0.04	1097	1105	1086	34.14	1219	1096	1101	0.76
70	A	1097	1133	1089	1088	2.87	1144	1095	1095	1.60	1095	1103	1084	13.58	1219	1096	1101	8.59
71	A	1097	1132	1089	1088	18.44	1133	1085	1085	24.26	1093	1101	1082	3.09	1212	1090	1095	1.17
72	A	1097	1130	1086	1085	12.78	1131	1083	1083	14.24	1093	1101	1082	4.60	1197	1077	1082	17.22
73	A	1049	1102	1060	1059	102.26	1102	1055	1056	90.41	1067	1075	1056	111.22	1194	1074	1079	108.32
74	A	1049	1099	1057	1056	61.96	1099	1052	1052	58.68	1063	1071	1052	76.01	1188	1068	1073	23.73
75	A	1039	1077	1035	1035	2.06	1079	1033	1034	3.84	1043	1050	1032	2.98	1151	1035	1039	1.26
76	A	1039	1069	1028	1027	10.41	1072	1026	1026	10.78	1035	1043	1024	10.04	1146	1030	1035	10.45
77	A	964	990	952	951	0.03	990	948	948	0.01	949	955	939	0.71	1119	1006	1011	0.42
78	A	952	981	943	943	1.06	982	940	940	0.98	948	955	938	0.44	1113	1001	1006	1.85
79	A	950	978	940	939	0.98	979	937	937	1.71	946	953	937	0.53	1092	982	987	0.01
80	A	950	977	939	939	0.67	978	936	936	1.11	937	944	927	1.52	1087	978	982	3.01
81	A	931	963	925	925	0.10	962	921	921	0.27	921	928	911	0.09	1049	944	948	0.16
82	A	929	957	920	919	5.25	956	915	915	7.15	919	925	909	6.37	1049	944	948	0.18
83	A	902	942	905	905	0.05	940	900	900	0.06	915	921	905	0.38	1018	915	919	0.04
84	A	902	942	905	905	2.95	940	900	900	7.03	912	919	902	2.65	1018	915	919	1.15
85	A	902	937	900	900	7.90	936	896	897	0.10	904	911	895	5.89	1014	912	916	10.84
86	A	902	934	898	897	0.09	936	896	896	2.10	898	905	889	0.00	1012	910	914	0.17
87	A	879	912	876	876	1.66	915	876	876	3.76	878	884	869	0.72	997	897	901	0.40
88	A	877	902	867	866	0.23	907	868	869	0.67	866	873	857	0.28	984	885	889	2.32
89	A	856	886	852	851	3.31	890	852	852	4.94	852	858	843	2.22	971	873	877	5.02
90	A	856	885	850	850	4.03	886	849	849	2.72	851	857	842	6.84	962	865	869	6.62
91	A	835	868	834	834	2.79	867	830	830	2.26	832	838	823	1.76	949	854	858	4.53
92	A	804	828	796	795	2.92	831	796	796	2.87	800	806	792	2.19	897	806	810	9.21
93	A	756	781	751	750	0.78	782	748	749	0.31	752	757	744	0.70	864	777	781	4.64
94	A	756	773	743	742	6.34	777	744	744	10.49	743	749	736	13.10	855	769	773	60.73
95	A	748	767	737	737	2.43	772	739	739	5.09	735	740	727	4.01	842	757	761	94.24
96	A	748	765	736	735	100.42	766	734	734	109.94	735	740	727	78.72	842	757	760	0.11
97	A	737	752	723	722	61.50	754	722	722	63.62	718	723	711	61.77	831	748	751	54.09
98	A	721	748	719	719	4.35	750	718	718	4.01	715	720	707	2.22	821	739	742	5.47
99	A	702	704	677	676	0.18	707	677	677	67.44	681	686	674	90.01	759	682	685	0.20
100	A	702	703	676	675	52.32	706	676	676	0.08	677	682	670	0.22	757	681	684	12.71
101	A	667	697	670	670	0.05	699	669	669	0.10	675	679	668	0.01	748	673	676	0.71
102	A	650	682	656	655	49.71	684	655	655	46.68	662	667	655	18.47	724	651	654	13.82
103	A	621	650	625	624	0.95	648	620	620	1.19	631	636	624	1.20	695	625	628	2.26
104	A	594	632	608	607	122.25	635	608	608	100.83	623	627	616	57.74	651	586	588	57.20
105	A	586	604	581	581	3.14	602	576	576	5.68	587	591	581	3.41	646	581	584	188.26
106	A	568	582	559	559	0.87	588	563	563	0.09	565	569	559	0.00	625	562	565	0.83
107	A	543	558	537	536	5.19	561	537	537	6.35	539	542	533	4.44	604	543	546	9.32
108	A	543	558	536	536	4.04	560	536	536	4.68	538	542	533	4.81	603	542	545	4.83
109	A	520	542	521	520	12.08	542	519	519	11.79	522	526	517	9.59	585	526	529	23.41
110	A	520	533	513	512	1.56	534	511	511	1.55	514	518	509	1.32	577	519	522	2.56
111	A	489	501	482	481	3.75	502	480	480	2.19	485	489	480	2.86	538	483	486	17.57
112	A	474	488	469	469	36.84	486	465	465	36.81	478	481	473	46.43	526	473	475	34.08
113	A	466	485	466	466	7.61	483	462	462	9.06	467	470	462	8.76	525	472	474	1.05
114	A	457	474	456	456	37.23	469	449	450	37.38	461	464	456	37.38	492	443	445	0.86
115	A	414	444	427	427	9.53	441	423	423	7.76	431	434	427	1.31	459	413	415	5.35
116	A	414	439	422	422	8.02	441	422	422	15.90	425	428	420	12.29	437	393	395	6.00
117	A	-	400	384	384	3.54	398	381	381	3.33	382	385	378	3.01	385	346	348	10.90
118	A	-	360	346	345	5.02	358	343	343	0.19	349	351	345	0.15	380	342	343	0.34
119	A	-	358	345	344	0.21	358	343	343	4.93	348	351	345	4.98	365	328	330	81.18
120	A	-	322	310	309	0.79	321	307	307	1.03	313	315	310	1.12	340	306	307	2.15
121	A	-	311	299	299	1.72	311	298	298	2.02	303	305	300	1.98	329	296	297	0.71
122	A	-	288	277	276	0.99	287	275	275	0.99	277	279	274	1.39	309	277	279	1.56
123	A	-	274	264	263	1.89	276	264	264	5.16	267	269	264	5.63	289	260	261	3.41
124	A	-	270	260	259	0.75	263	252	252	0.18	258	260	255	0.12	279	251	252	1.18
125	A	-	268	258	258	2.60	261	250	250	0.44	257	259	254	0.93	276	248	249	0.00
126	A	-	262	252	252	0.00	253	242	242	1.23	247	249	245	0.69	275	247	248	0.19
127	A	-	261	251	251	1.11	251	240	240	0.70	246	248	244	1.62	273	246	247	0.84
128	A	-	244	234	234	3.57	247	237	237	4.07	241	242	238	2.91	257	231	232	1.73
129	A	-	236	227	227	1.06	231	221	221	0.89	228	230	226	0.86	248	223	224	0.29
130	A	-	218	210	209	0.26	216	206	207	0.42	213	214	210	0.33	225	203	204	2.28
131	A	-	215	207	206	0.01	215	206	206	0.06	209	211	207	0.02	218	196	197	0.05
132	A	-	188	180	180	0.82	189	181	181	0.70	182	184	180	0.59	200	179	180	0.72
133	A	-	165	159	158	0.73	163	156	156	0.71	159	160	157	0.62	176	158	159	0.91
134	A	-	115	111	110	0.02	113	108	108	0.04	114	115	113	0.05	120	108	109	0.05
135	A	-	84	81	80	0.39	82	79	79	0.42	82	82	81	0.49	92	83	83	0.45
136	A	-	78	75	74	2.40	78	74	74	2.63	77	77	76	2.41	89	80	80	1.60
137	A	-	66	64	64	0.52	65	62	62	0.54	62	62	61	0.58	72	65	65	0.86
138	A	-	57	55	55	0.34	58	55	55	0.47	57	58	57	0.33	59	53	53	0.42
139	A	-	44	43	43	1.29	52	49	49	1.03	52	52	51	0.68	38	34	35	0.92
140	A	-	37	36	36	0.00	38	36	36	3.20	39	39	39	2.50	38	34	34	0.01
141	A	-	37	35	35	1.90	36	34	34	0.00	36	36	36	0.00	24	22	22	0.14
142	A	-	23	22	22	0.05	24	23	23	0.06	27	27	27	0.00	21	19	19	0.67
143	A	-	23	22	22	0.94	18	17	17	0.12	25	25	25	0.48	18	16	16	0.00
144	A	-	19	18	18	0.13	17	16	16	0.18	22	22	21	0.23	13	11	11	5.19

r	-	-	0.9999	0.9999	0.9999	-	0.9998	0.9998	0.9998	-	0.9998	0.9998	0.9998	-	0.9993	0.9993	0.9993	-
Mean absolute error	-	-	60.1664	10.7877	10.9685	-	64.0697	12.8383	12.7073	-	18.0533	25.3724	13.7327	-	167.1961	26.9652	27.3415	-
RMSov	-	-	64.9521	13.5503	13.4905	-	70.1891	14.5769	14.5704	-	22.5719	31.7346	15.4776	-	168.4846	31.8998	31.0622	-
RMSmol	-	-	72.6186	15.1497	15.0829	-	78.4738	16.2975	16.2902	-	25.2361	35.4804	17.3045	-	188.3715	35.6651	34.7286	-
Scaling factor (SF)	-	-	1.0000	0.9614	0.9606	-	1.0000	0.9573	0.9576	-	1.0000	1.0072	0.9895	-	1.0000	0.8992	0.9034	-

a Harmonic frequencies (in cm^−1^) and IR intensities (km/mol).

**Table 4S:** Optimized and experimental geometries of H_3_L^NNN^ in the ground state.

Bond lengths *	Bond lengths **	Exp. (Å)	Calculated, (Å)			

			B3LYP	B3PW91	BLYP	HF
C1-C2	C2-C3	1.383(3)	1.393	1.391	1.403	1.382
C1-C6	C3-C4	1.372(3)	1.395	1.393	1.405	1.384
C3-N21	C1-N1	1.394(3)	1.406	1.403	1.417	1.394
C3-C4	C1-C6	1.400(3)	1.418	1.415	1.431	1.403
C4-C5	C5-C6	1.384(3)	1.395	1.394	1.406	1.385
C4-N23	C6-N2	1.428(3)	1.431	1.424	1.442	1.423
C5-C6	C4-C5	1.387(3)	1.395	1.393	1.405	1.383
C11-C12	C11-C16	1.408(3)	1.418	1.415	1.431	1.403
C11-C13	C11-C12	1.399(3)	1.406	1.403	1.417	1.394
C11-N21	C11-N1	1.391(3)	1.390	1.385	1.400	1.388
C12-C14	C15-C16	1.377(3)	1.395	1.394	1.406	1.385
C12-N25	C16-N3	1.423(3)	1.431	1.424	1.442	1.423
C13-C15	C12-C13	1.375(3)	1.393	1.391	1.403	1.382
C14-C17	C14-C15	1.386(3)	1.395	1.393	1.405	1.383
C15-C17	C13-C14	1.380(3)	1.395	1.393	1.405	1.384
N23-C28	C7-N2	1.351(3)	1.393	1.388	1.409	1.373
N25-C27	C17-N3	1.356(3)	1.393	1.388	1.409	1.373
C27-C31	C17-C18	1.504(3)	1.536	1.531	1.550	1.529
C27-O49	C17-O2	1.231(3)	1.223	1.222	1.236	1.201
C28-C29	C7-C8	1.509(3)	1.536	1.531	1.550	1.529
C28-O50	C7-O1	1.228(3)	1.223	1.222	1.236	1.201
C29-C41	C8-C9	1.523(3)	1.541	1.536	1.554	1.535
C29-C45	C8-C10	1.512(3)	1.536	1.530	1.547	1.533
C31-C33	C18-C20	1.538(3)	1.541	1.536	1.554	1.535
C31-C37	C18-C19	1.519(3)	1.536	1.530	1.547	1.533

*r*			0.9906	0.9904	0.9888	0.9931

**Bond angles** *	**Bond angles** **	**Exp. (°)**	**Calculated (°)**			

			**B3LYP**	**B3PW91**	**BLYP**	**HF**

C2-C1-C6	C4-C3-C2	120.90(3)	120.88	120.86	120.86	120.83
C1-C2-C3	C3-C2-C1	120.60(2)	120.58	120.58	120.56	120.64
C2-C3-C4	C2-C1-C6	118.20(2)	118.45	118.49	118.48	118.55
C2-C3-N21	C2-C1-N1	123.80(2)	123.83	123.70	123.88	122.96
C4-C3-N21	N1-C1-C6	117.90(2)	117.64	117.73	117.56	118.44
C3-C4-C5	C5-C6-C1	120.60(3)	119.99	119.99	119.97	119.81
C3-C4-N23	C1-C6-N2	118.40(2)	118.45	118.31	118.22	119.17
C5-C4-N23	C5-C6-N2	121.00(2)	121.46	121.61	121.72	120.84
C4-C5-C6	C6-C5-C4	120.40(3)	121.04	121.01	120.97	121.31
C1-C6-C5	C3-C4-C5	119.30(2)	119.00	119.02	119.13	118.79
C12-C11-C13	C12-C11-C16	118.20(2)	118.45	118.49	118.48	118.55
C12-C11-N21	N1-C11-C16	118.40(2)	117.64	117.73	117.56	118.44
C13-C11-N21	N1-C11-C12	123.40(2)	123.83	123.70	123.88	122.96
C11-C12-C14	C15-C16-C11	120.10(2)	119.99	119.99	119.97	119.81
C11-C12-N25	C11-C16-N3	118.80(2)	118.45	118.31	118.22	119.17
C14-C12-N25	C15-C16-N3	121.10(2)	121.46	121.61	121.72	120.84
C11-C13-C15	C13-C12-C11	120.60(2)	120.58	120.58	120.56	120.64
C12-C14-C17	C16-C15-C14	121.20(2)	121.04	121.01	120.97	121.31
C13-C15-C17	C12-C13-C14	121.20(2)	120.88	120.86	120.86	120.83
C14-C17-C15	C13-C14-C15	118.70(2)	119.00	119.02	119.13	118.79
C3-N21-C11	C11-N1-C1	130.00(2)	130.62	130.06	130.86	129.57
C4-N23-C28	C7-N2-C6	125.90(2)	130.28	129.95	130.63	132.35
C12-N25-C27	C17-N3-C16	124.50(2)	130.28	129.95	130.63	132.34
N25-C27-C31	N3-C17-C18	115.70(2)	121.15	121.15	121.23	122.27
N25-C27-O49	O2-C17-N3	121.80(2)	118.54	118.64	118.44	118.05
C31-C27-O49	O2-C17-C18	122.40(2)	120.22	120.12	120.21	119.64
N23-C28-C29	N2-C7-C8	115.20(2)	121.15	121.15	121.23	122.27
N23-C28-O50	O1-C7-N2	121.90(3)	118.54	118.64	118.44	118.04
C29-C28-O50	O1-C7-C8	122.90(2)	120.22	120.12	120.21	119.64
C28-C29-C41	C7-C8-C9	110.30(2)	111.74	111.27	111.29	113.42
C28-C29-C45	C7-C8-C10	112.00(2)	115.88	116.55	116.69	114.03
C41-C29-C45	C10-C8-C9	110.40(2)	112.13	112.21	112.26	112.10
C27-C31-C33	C17-C18-C20	108.30(2)	111.74	111.27	111.29	113.42
C27-C31-C37	C17-C18-C19	111.20(2)	115.88	116.55	116.69	114.03
C33-C31-C37	C19-C18-C20	111.90(2)	112.13	112.21	112.26	112.10

*r*			0.8716	0.8686	0.8589	0.8175

* The atom-numbering scheme of the molecular structure is given in Figure 4.

** The atom-numbering scheme of the molecular structure is given in Figure 1a.

## Figures and Tables

**Scheme:**
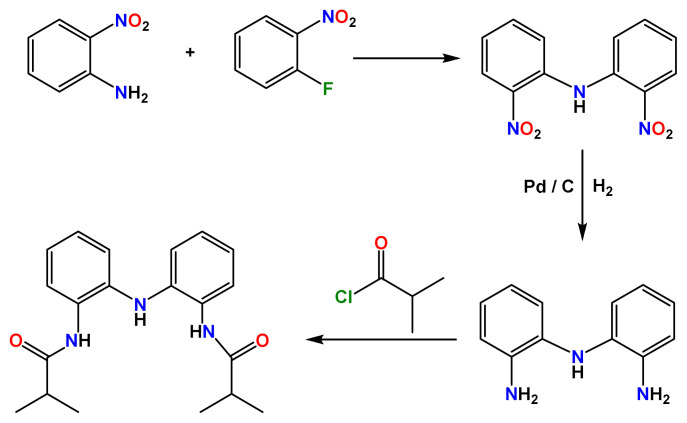
Synthesis of H_3_L^NNN^.

**Table 1:** Crystal data and structure refinement for H_3_L^NNN^.

Empirical formula	C_20_H_25_N_3_O_2_
Formula weight	339.43
Temperature (K)	173(2)
Crystal system	Triclinic
Space group	*P*-1
a (Å)	9.5377(9)
b (Å)	10.9710(10)
c (Å)	18.6693(15)
α (°)	76.644(6)
β (°)	80.010(6)
γ (°)	81.379(7)
Volume (Å^3^)	1859.5(3)
Z	4
ρ_calc_ (mg/mm^3^)	1.212
m (mm^−1^)	0.633
F(000)	728.0
Crystal size (mm^3^)	0.35 × 0.06 × 0.03
2Θ range for data collection	8.338 to 130.168°
Index ranges	−11 ≤ *h* ≤ 11, −13 ≤ *k* ≤ 12, −21 ≤ *l* ≤ 17
Reflections collected	15409
Independent reflections	5785 [*R*_int_ = 0.0490, *R*_sigma_ = 0.1053]
Data/restraints/parameters	5785/0/452
Goodness-of-fit on *F*^2^	1.009
Final R indexes [I ≥ 2σ (I)]	R_1_ = 0.0599, wR_2_ = 0.1390
Final R indexes [all data]	R_1_ = 0.1095, wR_2_ = 0.1636
Largest diff. peak/hole (e.Å^−3^)	0.24/−0.23

**Table 2:** Selected bond lengths for H_3_L^NNN^.*

Atom	Atom	Length (Å)	Atom	Atom	Length (Å)
C1	C2	1.394(3)	C1B	C2B	1.391(3)
C1	C6	1.400(3)	C1B	C6B	1.403(3)
C1	N1	1.394(3)	C1B	N1B	1.395(3)
C2	C3	1.383(3)	C2B	C3B	1.380(3)
C3	C4	1.372(3)	C3B	C4B	1.383(4)
C4	C5	1.387(3)	C4B	C5B	1.387(3)
C5	C6	1.384(3)	C5B	C6B	1.377(3)
C6	N2	1.428(3)	C6B	N2B	1.429(3)
C7	C8	1.509(3)	C7B	C8B	1.512(3)
C7	N2	1.351(3)	C7B	N2B	1.335(3)
C7	O1	1.228(3)	C7B	O1B	1.227(3)
C8	C9	1.523(3)	C8B	C9B	1.531(3)
C11	N1	1.391(3)	C11B	N1B	1.395(3)
C16	N3	1.423(3)	C16B	N3B	1.434(3)
C17	N3	1.356(3)	C17B	N3B	1.362(3)

* The atom-numbering scheme of the molecular structure is given in Figure 1a.

**Table 3:** Selected bond angles for H_3_L^NNN^.*

Atom	Atom	Atom	Angle (°)	Atom	Atom	Atom	Angle (°)
C2	C1	C6	118.2(2)	C2B	C1B	C6B	118.1(2)
C2	C1	N1	123.8(2)	C2B	C1B	N1B	123.6(2)
N1	C1	C6	117.9(2)	N1B	C1B	C6B	118.2(2)
C3	C2	C1	120.6(2)	C3B	C2B	C1B	120.7(2)
C4	C3	C2	120.9(3)	C2B	C3B	C4B	120.9(3)
C3	C4	C5	119.3(2)	C3B	C4B	C5B	118.9(3)
C5	C6	N2	121.0(2)	C5B	C6B	N2B	120.1(2)
N2	C7	C8	115.2(2)	N2B	C7B	C8B	116.4(2)
O1	C7	C8	122.9(2)	O1B	C7B	C8B	122.3(3)
O1	C7	N2	121.9(3)	O1B	C7B	N2B	121.3(3)
N1	C11	C12	123.4(2)	C12B	C11B	N1B	123.9(2)
N1	C11	C16	118.4(2)	N1B	C11B	C16B	118.2(2)
N3	C17	C18	115.7(2)	N3B	C17B	C18B	115.7(2)
O2	C17	N3	121.8(2)	O2B	C17B	N3B	121.7(3)
C11	N1	C1	130.0(2)	C11B	N1B	C1B	128.9(2)
C7	N2	C6	125.9(2)	C7B	N2B	C6B	122.7(2)
C17	N3	C16	124.5(2)	C17B	N3B	C16B	123.7(2)

* The atom-numbering scheme of the molecular structure is given in Figure 1a.

**Table 4:** Hydrogen bonds for the title compound (Å, °).*

D	H	A	d(H···A)	d(D···A)	∠ D-H···A
N2	H2A	O1B i	1.89	2.767(3)	176
N3	H3A	O2B ii	1.99	2.860(3)	171
N2B	H2BA	O1 ii	1.93	2.806(3)	176
N3B	H3BA	O2 iii	2.00	2.867(3)	167
C18	H18	O2B ii	2.54	3.324(3)	135
**D**	**H**	**Cg**	**d(H···Cg)**	**d(D···Cg)**	**∠ D-H···Cg**
C10	H10C	Cg1 ii	3.7790(11)	4.529(3)	35.87(13)
C9	H9B	Cg1 ii	3.0263(10)	3.933(3)	6.16(4)
C9B	H9BA	Cg2 iii	3.300(1)	6.370(3)	121.60(17)
C9B	H9BB	Cg2 iii	3.7447(10)	6.370(3)	92.00(16)

* Symmetry codes:

i 1+*x*, +*y*, +*z*;

ii *x*, *y*, *z*;

iii −1+*x*, +*y*, +*z*.

Cg1 is the centroid of C11B, C16B, C15B, C14B, C13B and C12B; Cg2 is the centroid of C1, C6, C5, C4, C3 and C2.

**Table 5:** Selected optimized and experimental geometries of H_3_L^NNN^ in the ground state.*

Bond lengths	Exp., (Å)	Calculated, (Å)			
		B3LYP	B3PW91	BLYP	HF
C2-C3	1.383(3)	1.393	1.391	1.403	1.382
C3-C4	1.372(3)	1.395	1.393	1.405	1.384
C1-N1	1.394(3)	1.406	1.403	1.417	1.394
C1-C6	1.400(3)	1.418	1.415	1.431	1.403
C5-C6	1.384(3)	1.395	1.394	1.406	1.385
C6-N2	1.428(3)	1.431	1.424	1.442	1.423
C4-C5	1.387(3)	1.395	1.393	1.405	1.383
C11-C16	1.408(3)	1.418	1.415	1.431	1.403
C11-N1	1.391(3)	1.390	1.385	1.400	1.388
C15-C16	1.377(3)	1.395	1.394	1.406	1.385
C13-C14	1.380(3)	1.395	1.393	1.405	1.384
C7-N2	1.351(3)	1.393	1.388	1.409	1.373
C17-N3	1.356(3)	1.393	1.388	1.409	1.373
*r*		0.9906	0.9904	0.9888	0.9931
**Bond angles**	**Exp. (°)**	**Calculated (°)**			
		**B3LYP**	**B3PW91**	**BLYP**	**HF**
C4-C3-C2	120.90(3)	120.88	120.86	120.86	120.83
C3-C2-C1	120.60(2)	120.58	120.58	120.56	120.64
C2-C1-C6	118.20(2)	118.45	118.49	118.48	118.55
C2-C1-N1	123.80(2)	123.83	123.70	123.88	122.96
N1-C1-C6	117.90(2)	117.64	117.73	117.56	118.44
C5-C6-C1	120.60(3)	119.99	119.99	119.97	119.81
C1-C6-N2	118.40(2)	118.45	118.31	118.22	119.17
C5-C6-N2	121.00(2)	121.46	121.61	121.72	120.84
C6-C5-C4	120.40(3)	121.04	121.01	120.97	121.31
C3-C4-C5	119.30(2)	119.00	119.02	119.13	118.79
C12-C11-N1	123.40(2)	123.83	123.70	123.88	122.96
C11-C16-N3	118.80(2)	118.45	118.31	118.22	119.17
C15-C16-N3	121.10(2)	121.46	121.61	121.72	120.84
C11-N1-C1	130.00(2)	130.62	130.06	130.86	129.57
C7-N2-C6	125.90(2)	130.28	129.95	130.63	132.35
C17-N3-C16	124.50(2)	130.28	129.95	130.63	132.34
N3-C17-C18	115.70(2)	121.15	121.15	121.23	122.27
O2-C17-N3	121.80(2)	118.54	118.64	118.44	118.05
O2-C17-C18	122.40(2)	120.22	120.12	120.21	119.64
*r*		0.8716	0.8686	0.8589	0.8175

* The atom-numbering scheme of the molecular structure is given in Figure 1a.

**Table 6:** The calculated thermodynamic parameters of H_3_L^NNN^.

Thermodynamic parameters (298 K)	B3LYP	B3PW91	BLYP	HF
SCF energy (a.u.)	−1091.960	−1091.550	−1091.487	−1085.070
Total energy (Thermal) *E*_total_ (kcal/mol)	280.443	281.057	272.755	297.750
Heat capacity at const. volume, C_v_ (cal/mol.K)	95.000	94.973	97.989	89.040
Vibrational energy, *E*_vib_ (kcal/mol)	278.666	279.279	270.978	295.973
Zero-point vibrational energy, *E*_o_ (kcal/mol)	264.79348	265.37380	256.69694	282.80463
Rotational constant (GHz)				
A	0.29458	0.29799	0.28888	0.29425
B	0.16631	0.16172	0.15820	0.17748
C	0.12168	0.11928	0.11607	0.12799
Dipole moment (Debye)				
μ_x_	0.0000	0.0000	0.0000	−0.0001
μ_y_	5.2655	5.1142	4.8185	5.9072
μ_z_	0.0000	0.0000	−0.0001	−0.0001
μ_Total_	5.2655	5.1142	4.8185	5.9072
Entropy (cal/mol.K)				
Translational	43.359	43.359	43.359	43.359
Rotational	35.243	35.279	35.359	35.130
Vibrational	97.066	97.826	98.620	95.191
Total	175.668	176.464	177.338	173.679

**Table 7:** Vibrational wavenumbers obtained for H_3_L^NNN^ at B3LYP/6-31G(d,p) level.^a^

No.	Exp.	Wavenumber			IR intensity	Assignments, PED (%) b
		Unscaled	Scaled	Scaled		
1	3406	3589	3451	3448	63.43	100 ν(N_1_-H)
2	3398	3541	3404	3401	0.47	100 ν(N_2,3_-H)
3	3367	3541	3404	3401	35.13	100 ν(N_2,3_-H)
4	3118	3232	3107	3105	5.33	94 ν(CH), sym, Ar-H
5	3118	3223	3099	3096	1.42	92 ν(CH), sym, Ar-H
6	3115	3215	3091	3088	2.10	97 ν(CH), sym, Ar-H
7	3115	3214	3090	3088	33.69	97 ν(CH), sym, Ar-H
8	3099	3201	3077	3075	21.96	98 ν(CH), asym, Ar-H
9	3099	3201	3077	3075	2.03	97 ν(CH), asym, Ar-H
10	3059	3189	3066	3063	5.58	92 ν(CH), asym, Ar-H
11	3059	3188	3065	3063	3.25	90 ν(CH), asym, Ar-H
12	3035	3141	3019	3017	63.58	87 ν(CH_3_), asym
13	3035	3140	3019	3017	1.78	87 ν(CH_3_), asym
14	3001	3135	3014	3012	17.46	95 ν(CH_3_), asym
15	3001	3135	3014	3012	8.52	95 ν(CH_3_), asym
16	2966	3120	2999	2997	41.55	98 ν(CH_3_), asym
17	2966	3120	2999	2997	6.51	98 ν(CH_3_), asym
18	2964	3112	2992	2990	4.55	98 ν(CH_3_), asym
19	2964	3112	2992	2989	51.28	98 ν(CH_3_), asym
20	2958	3072	2954	2951	7.76	95 ν(CH)
21	2958	3072	2954	2951	0.32	95 ν(CH)
22	2938	3051	2934	2931	0.02	99 ν(CH_3_), sym
23	2938	3051	2934	2931	31.13	99 ν(CH_3_), sym
24	2929	3049	2931	2928	52.46	98 ν(CH_3_), sym
25	2929	3049	2931	2928	1.60	99 ν(CH_3_), sym
26	1695	1776	1707	1706	495.87	82 ν(C=O)
27	1679	1773	1704	1703	139.36	82 ν(C=O)
28	1608	1662	1597	1596	2.25	66 ν(C=C)
29	1597	1649	1585	1584	200.24	66 ν(C=C)
30	1579	1637	1574	1573	111.12	54 ν(C=C) + 18 δ(CNH)
31	1568	1628	1565	1564	0.69	62 ν(C=C)
32	1527	1574	1513	1512	486.94	50 ν(C=C) + 17 δ(CNH)
33	1490	1534	1474	1473	84.39	35 ν(C=C) + 17 δ_d_(CH_3_)
34	1472	1530	1471	1469	5.69	81 δ(CH_3_), deform.
35	1472	1529	1470	1469	30.21	89 δ(CH_3_), deform.
36	1458	1522	1463	1462	1.55	89 δ(CH_3_), deform.
37	1458	1521	1463	1461	29.05	83 δ(CH_3_), deform.
38	1454	1513	1455	1454	0.02	90 δ(CH_3_), deform.
39	1454	1513	1455	1453	0.74	90 δ(CH_3_), deform.
40	1447	1506	1448	1447	0.08	70 δ(CH_3_), deform.
41	1446	1506	1448	1447	2.97	83 δ(CH_3_), deform.
42	1446	1505	1447	1445	0.44	50 δ(CH_3_), deform. + 19 δ(CCH)
43	1436	1498	1440	1439	101.95	66 δ(CCH) + 24 ν(C=C)
44	1421	1474	1417	1416	31.67	29 δ(CNH) + 11 ν(C=C) + 10 ν(NC) + 10 δ(CCH)
45	1406	1440	1385	1383	41.14	50 δ(CNH) + 15 ν(NC)
46	1392	1440	1384	1383	38.26	50 δ(CNH) + 16 δ(CH_3_), umbrella
47	1382	1435	1380	1378	2.34	55 δ(CH_3_) umbrella + 17 δ(CNH)
48	1382	1433	1378	1377	4.49	46 δ(CH_3_) umbrella + 23 δ(CNH )
49	1357	1411	1356	1355	2.07	69 δ(CH_3_) umbrella
50	1357	1411	1356	1355	6.47	69 δ(CH_3_) umbrella
51	1325	1375	1322	1321	107.77	34 ν(CN) + 30 δ(CH_3_) + 16 ν(C=C)
52	1307	1360	1308	1307	0.29	65 δ(CCH)
53	1307	1360	1308	1307	6.14	71 δ(CCH)
54	1305	1357	1305	1304	113.42	44 δ(CCH) + 13 ν(NC)
55	1305	1354	1302	1301	65.15	65 δ(CCH)
56	1288	1348	1296	1295	5.51	61 ν(C=C) + 15 δ(CCH)
57	1286	1338	1286	1285	303.84	40 ν(CC) + 19 ν(CN)
58	1286	1335	1283	1282	2.94	53 δ(CCH ) + 18 ν(C=C)
59	1263	1333	1281	1280	164.54	50 ν(CC) + 29 ν(CN)
60	1263	1321	1270	1269	0.52	39 ν(C=C) + 28 δ(CCH)
61	1249	1288	1238	1237	16.47	26 ν(C=C) + 18 δ(CCH) + 17 ν(CN)
62	1213	1279	1230	1229	3.52	34 ν(CN) + 20 ν(C=C) + 18 δ(CCH)
63	1195	1227	1179	1178	4.51	25 δ(CCH) + 20 ν(CN) + 10 ν(C=C)
64	1126	1222	1174	1174	3.38	21 δ(CCH) + 15 ν(CN) + 14 ν(C=C)
65	1109	1197	1150	1150	16.2	51 δ(CH_3_), rocking
66	1109	1197	1150	1149	5.34	50 δ(CH_3_), rocking
67	1109	1191	1145	1144	1.83	72 δ(CCH)
68	1109	1189	1143	1142	3.53	73 δ(CCH)
69	1097	1133	1090	1089	10.33	40 ν(CC) + 10 δ(CCH)
70	1097	1133	1089	1088	2.87	46 ν(CC) + 10 δ(CCH)
71	1097	1132	1089	1088	18.44	34 ν(CC) + 10 δ(CCH)
72	1097	1130	1086	1085	12.78	38 ν(C=C) + 10 δ(CCH)
73	1049	1102	1060	1059	102.26	37 δ(CH_3_), rocking + 12 ν(CN)
74	1049	1099	1057	1056	61.96	35 δ(CH_3_), rocking + 12 ν(CN)
75	1039	1077	1035	1035	2.06	59 ν(C=C) + 10 δ(CCH), Ring breathing
76	1039	1069	1028	1027	10.41	67 ν(C=C)
77	964	990	952	951	0.03	74 γ(CH)
78	952	981	943	943	1.06	63 γ(CH)
79	950	978	940	939	0.98	34 δ(CCH) + 30 ν(CC)
80	950	977	939	939	0.67	30 δ(CCH) + 27 ν(CC)
81	931	963	925	925	0.10	73 γ(CH)
82	929	957	920	919	5.25	75 γ(CH)
83	902	942	905	905	0.05	66 δ(CH_3_), rocking
84	902	942	905	905	2.95	65 δ(CH_3_), rocking
85	902	937	900	900	7.90	31 δ(CCC) + 16 ν(C=C)
86	902	934	898	897	0.09	40 ν(CC)
87	879	912	876	876	1.66	37 ν(CC) + 10 δ(CCC)
88	877	902	867	866	0.23	40 ν(CC) + 15 γ(CH)
89	856	886	852	851	3.31	42 γ(CH)
90	856	885	850	850	4.03	31 ν(C=C) + 10 ν(CC), Ring breathing
91	835	868	834	834	2.79	54 γ(CH)
92	804	828	796	795	2.92	21 ν(C=C) + 13 ν(CN) + 10 δ(CCC) + 10 δ(CNC)
93	756	781	751	750	0.78	55 γ(CH)
94	756	773	743	742	6.34	28 γ(CH) + 10 ν(CC)
95	748	767	737	737	2.43	41 γ(CO) + 18 γ(CH)
96	748	765	736	735	100.42	31 γ(CH)
97	737	752	723	722	61.50	41 γ(CO) + 16 γ(CH) + 13 γ(C)
98	721	748	719	719	4.35	41 γ(CO) + 16 γ(CH) + 12 γ(C)
99	702	704	677	676	0.18	13 ν(CC) + 14 δ(CCC) + 12 ν(NC) + 11 δ(CNC)
100	702	703	676	675	52.32	22 ν(CC) + 10 ν(NC) + 11 δ(CCC)
101	667	697	670	670	0.05	17 δ(CNC) + 10 δ(CCN) + 10 γ(NH)
102	650	682	656	655	49.71	16 δ(CCC) + 10 γ(NH)
103	621	650	625	624	0.95	25 δ(CCC) + 12 ν(C=C) + 10 δ(CCN)
104	594	632	608	607	122.25	48 γ(NH)
105	586	604	581	581	3.14	65 δ(CCC)
106	568	582	559	559	0.87	59 γ(NH)
107	543	558	537	536	5.19	34 γ(C) + 10 δ(CCC)
108	543	558	536	536	4.04	36 γ(C) + 10 δ(CCC)
109	520	542	521	520	12.08	30 ν(CC) + 15 δ(CCC)
110	520	533	513	512	1.56	20 ν(CC) + 16 δ(CCC) + 10 δ(CCN)
111	489	501	482	481	3.75	40 δ(CCN) + 10 δ(OCN)
112	474	488	469	469	36.84	40 γ(NH) + 23 γ(C)
113	466	485	466	466	7.61	67 γ(C)
114	457	474	456	456	37.23	49 γ(NH) + 10 δ(OCN)
115	414	444	427	427	9.53	41 δ(CCN)
116	414	439	422	422	8.02	20 δ(CCN) + 11 ν(NC) + 10 δ(CCO)
117	-	400	384	384	3.54	11 ν(NC) + 10 δ(CCN) + 10 δ(CCO) + 10 δ(OCN)
118	-	360	346	345	5.02	28 δ((H_3_C)-C-(CH_3_))
119	-	358	345	344	0.21	32 δ((H_3_C)-C-(CH_3_))
120	-	322	310	309	0.79	32 δ((H_3_C)-C-(CH_3_))
121	-	311	299	299	1.72	23 δ((H_3_C)-C-(CH_3_)) + 13 γ(C)
122	-	288	277	276	0.99	21 δ(NCC) + 18 γ(Ph-N-Ph)
123	-	274	264	263	1.89	52 τ(CH_3_)
124	-	270	260	259	0.75	87 τ(CH_3_)
125	-	268	258	258	2.60	33 τ(CH_3_)
126	-	262	252	252	0.10	77 τ(CH_3_)
127	-	261	251	251	1.11	56 τ(CH_3_) + 10 δ(CCN)
128	-	244	234	234	3.57	43 δ(NCC)
129	-	236	227	227	1.06	34 τ(CH_3_) + 20 δ(CCC) + 10 δ(CCN)
130	-	218	210	209	0.26	58 τ(CH_3_) + 30 δ(CCC)
131	-	215	207	206	0.01	11 δ(CCC) + 10 ν(NC) + 15 τ(CN)
132	-	188	180	180	0.82	10 δ(CCN) + 10 τ(CC) + 20 τ(CN)
133	-	165	159	158	0.73	22 δ(CCN) + 20 τ(CC) + 11 τ(CN)
134	-	115	111	110	0.02	32 δ(CNC) + 13 τ(CC) + 12 τ(CN)
135	-	84	81	80	0.39	28 δ(CNC) + 23 τ(CC)
136	-	78	75	74	2.40	53 τ(CN)
137	-	66	64	64	0.52	48 τ(NC) + 20 τ(CC)
138	-	57	55	55	0.34	59 τ(CN)
139	-	44	43	43	1.29	71 τ(CN) + 16 τ(CC)
140	-	37	36	36	0.10	18 τ(OCNC) + 16 τ(CNCCH_3_) + 10 δ(CNC)
141	-	37	35	35	1.90	58 τ(CNCCH_3_) + 26 τ(CC) + 14 τ(CCCO)
142	-	23	22	22	0.05	48 τ(CNCCH_3_) + 21 τ(HNCC) + 12 τ(CCCO)
143	-	23	22	22	0.94	48 τ(HNCCH_3_) + 40 τ(CNCC)
144	-	19	18	18	0.13	53 τ(CN) + 47 τ(CCCO)
*r*	0.9999	0.9999	0.9999			
Mean absolute percentage error	3.9443	0.8635	0.8833			
RMS_over_	64.9521	13.5503	13.4905			
RMS_mol_	72.6186	15.1497	15.0829			
Scaling factor	1.0000	0.9614	0.9606			

a Harmonic frequencies (in cm^−1^) and IR intensities (km/mol).

b ν, stretching; δ, in-plane bending; γ, out-of-plane bending; τ, torsion; sym, symmetric; asym, asymmetric; deform., deformation; PED less than 10% are not shown.

## Data Availability

Sample of the compound is available from the author.
