# Supplementary figures and images for: Crystal structure and vibrational spectra of bis(2-isobutyrylamidophenyl)amine: a redox noninnocent ligand
Source: Turk J Chem. 2021 Sep 12;45(6):1933–51. doi: 10.3906/kim-2106-56 (PMC10734769; doi:10.3906/kim-2106-56)

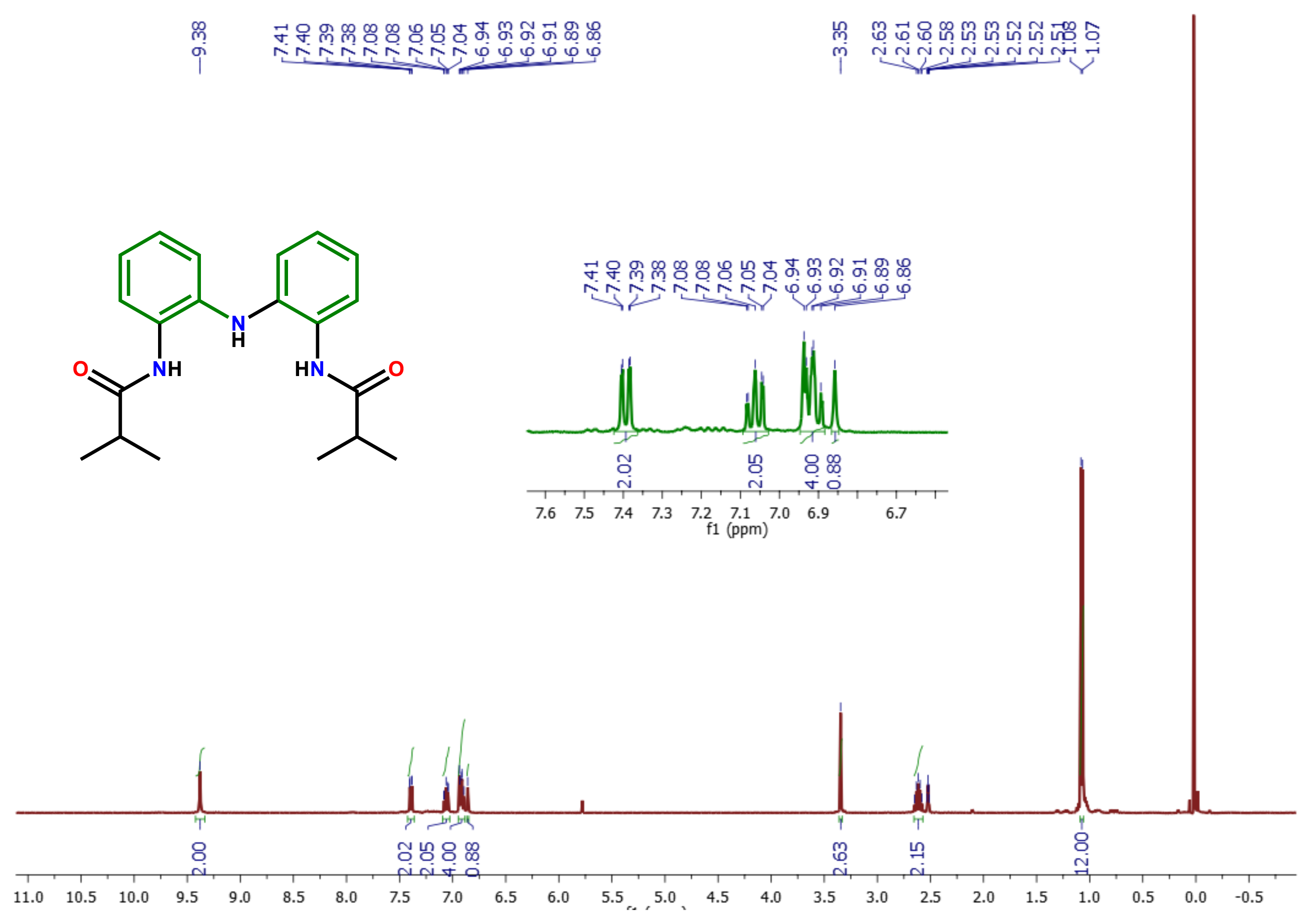

Supplement: Figure 1S — 1H NMR spectra of H3LNNN in DMSO-d6. [file turkjchem-45-6-1933s1.tif]

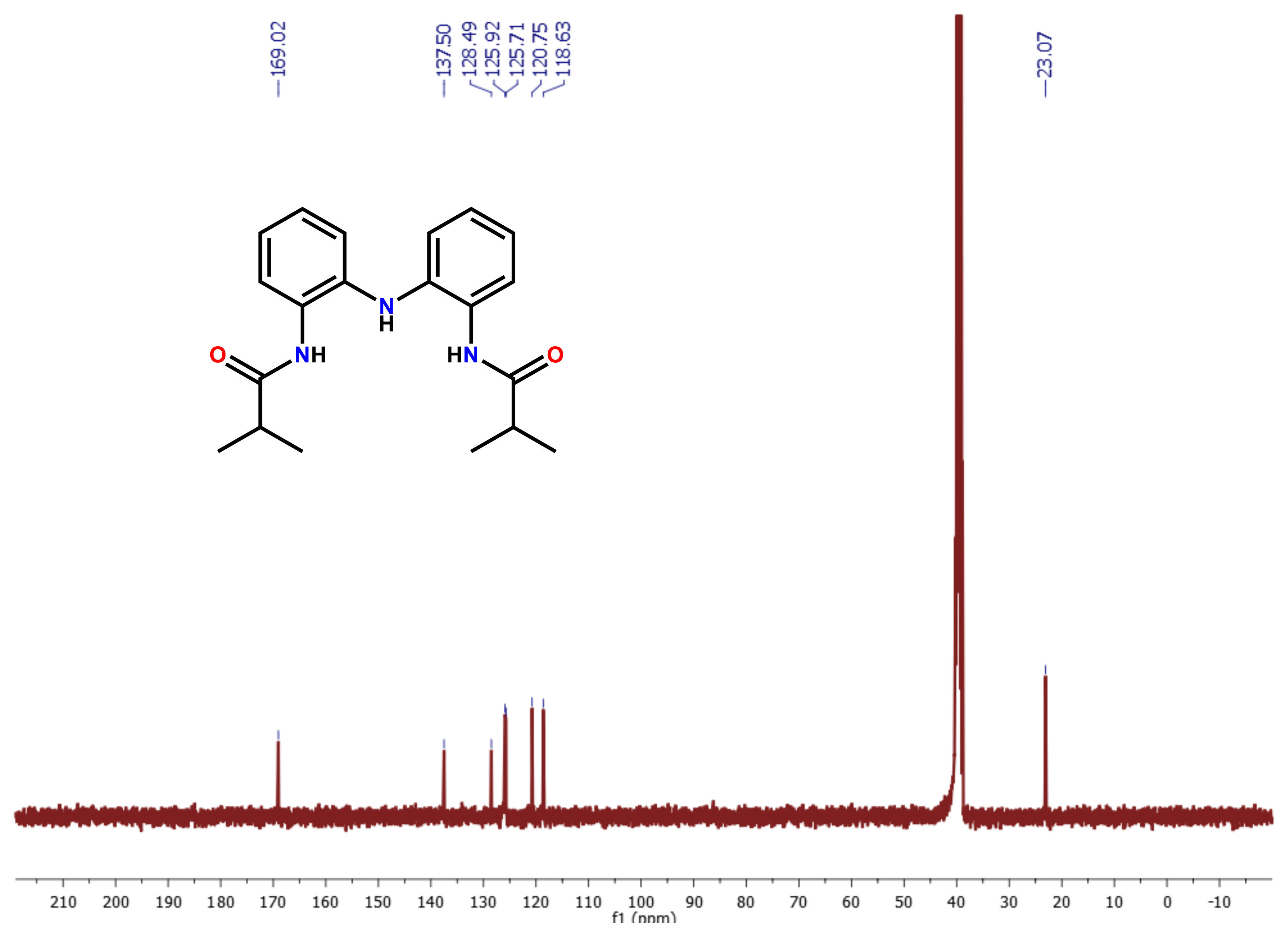

Supplement: Figure 2S — 13C NMR spectra of H3LNNN in DMSO-d6. [file turkjchem-45-6-1933s2.tif]

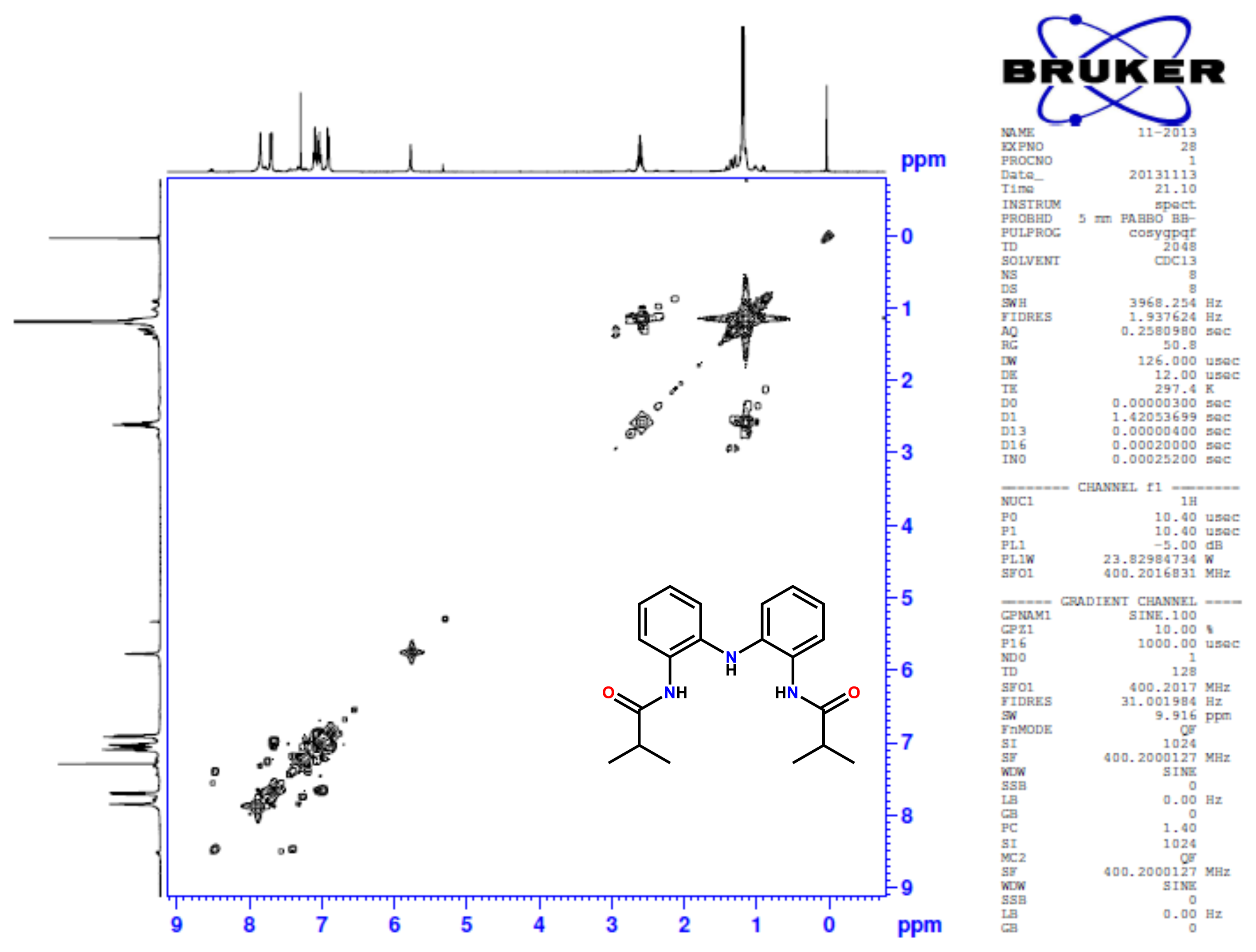

Supplement: Figure 3S — COSY-NMR spectra of H3LNNN in DMSO-d6. [file turkjchem-45-6-1933s3.tif]

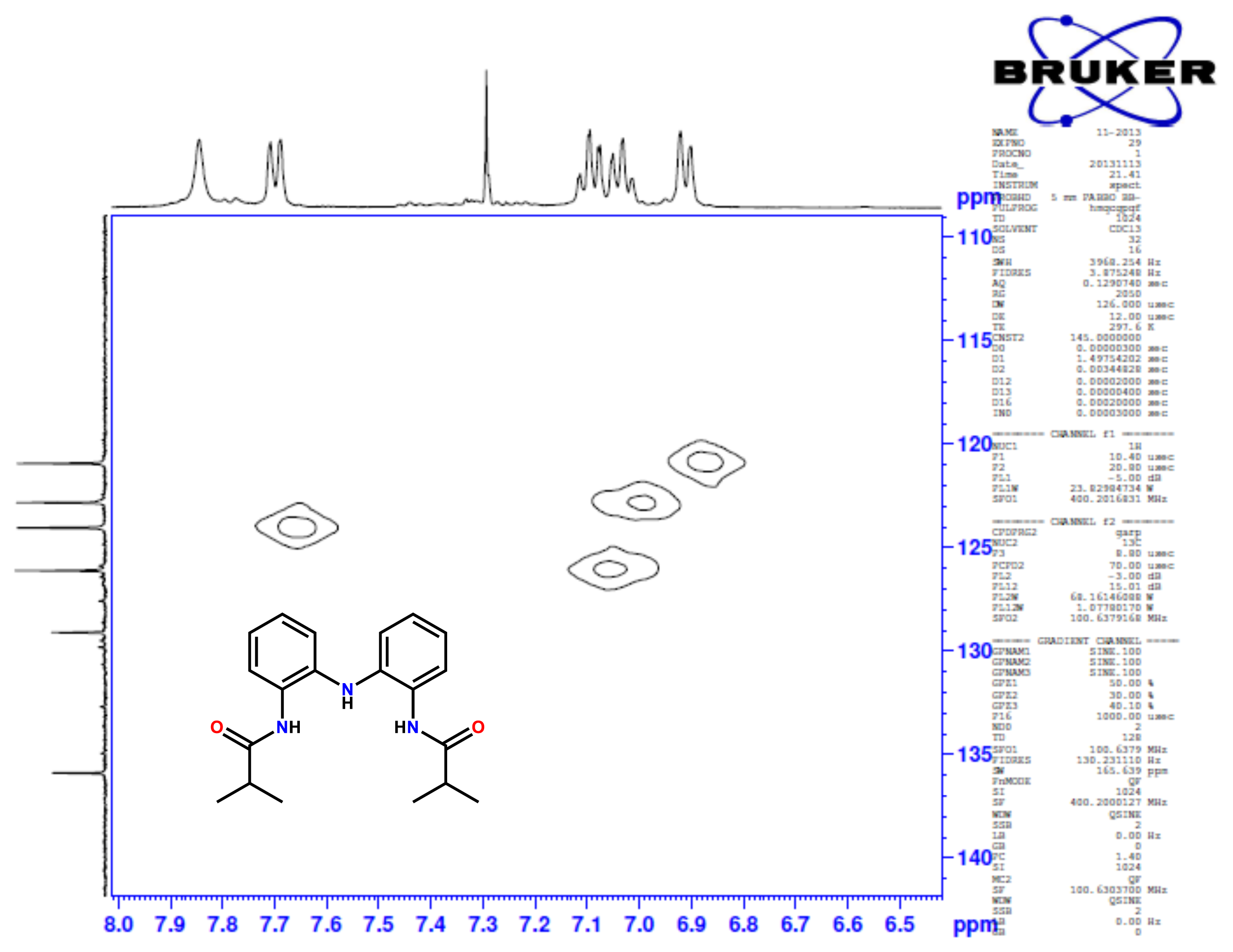

Supplement: Figure 4S — HMQC-NMR spectra of H3LNNN in DMSO-d6. [file turkjchem-45-6-1933s4.tif]

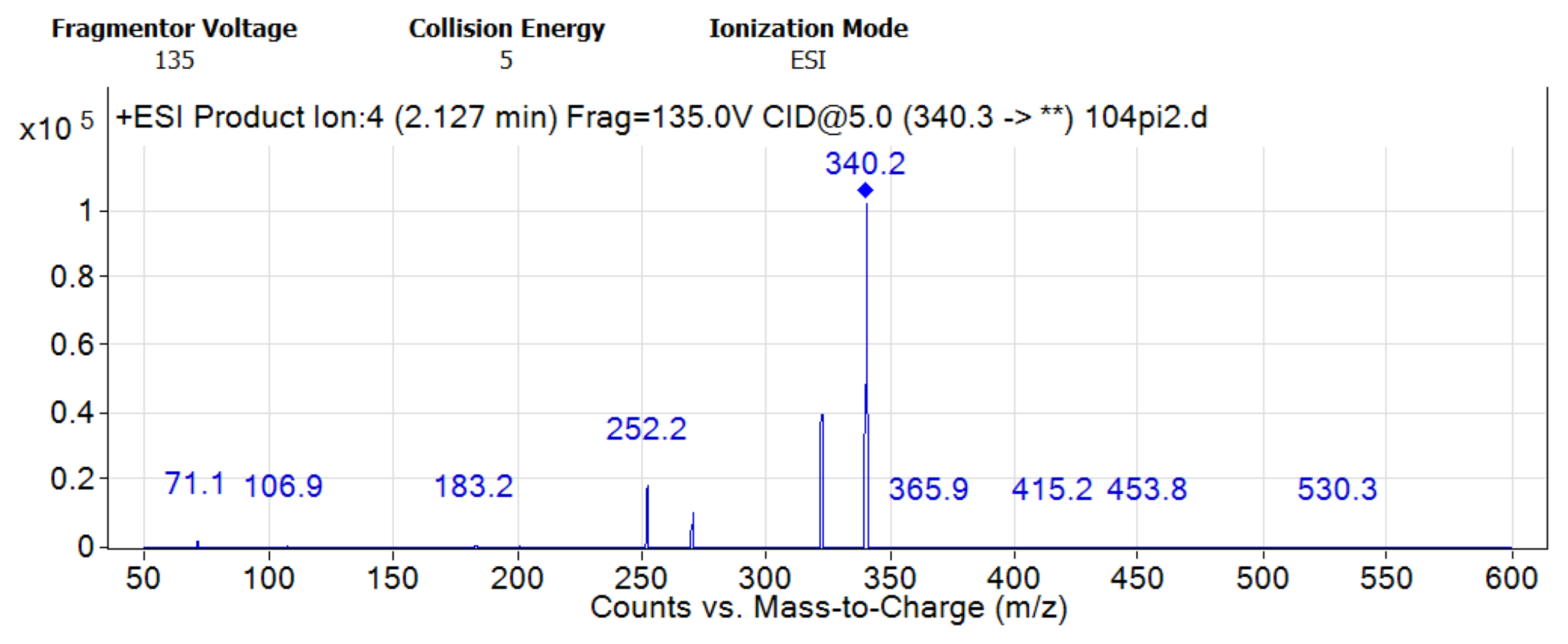

Supplement: Figure 5S — LC-MS spectra of H3LNNN. [file turkjchem-45-6-1933s5.tif]

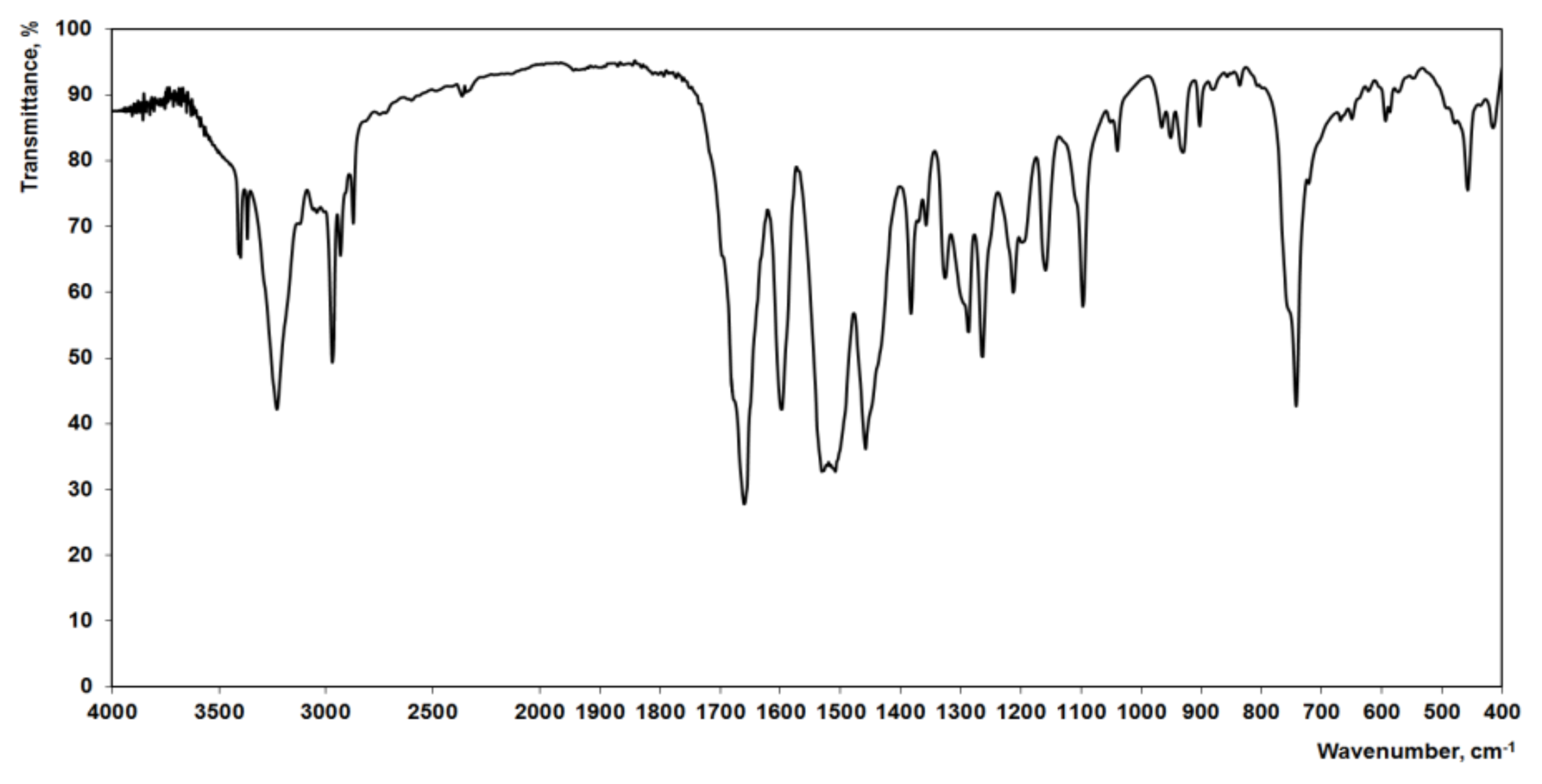

Supplement: Figure 6S — FT-IR spectrum of H3LNNN. [file turkjchem-45-6-1933s6.tif]

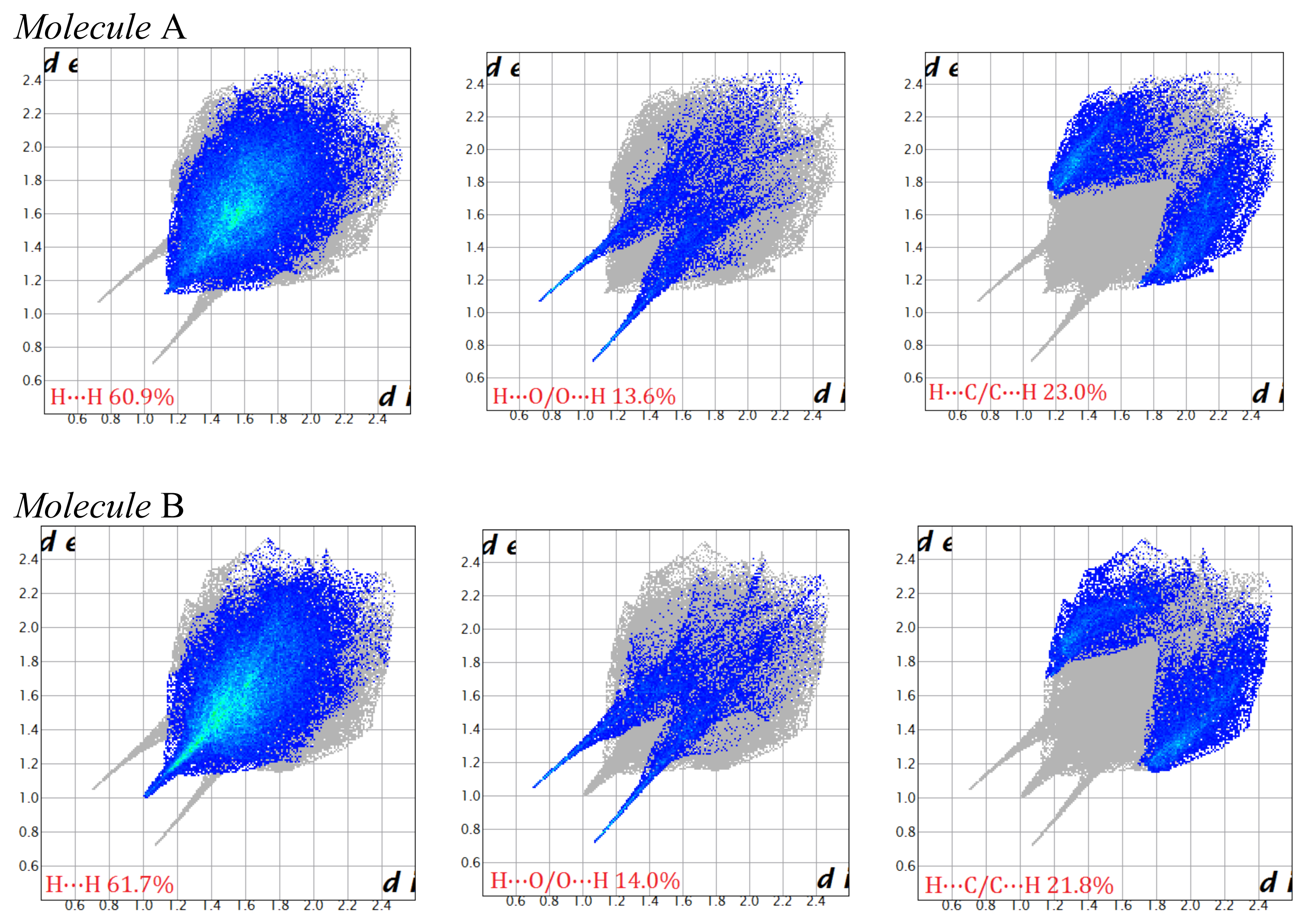

Supplement: Figure 7S — 2D fingerprint plots of molecules A and B. [file turkjchem-45-6-1933s7.tif]
